# Conformational rearrangements in the transmembrane domain of CNGA1 channels revealed by single-molecule force spectroscopy

**DOI:** 10.1038/ncomms8093

**Published:** 2015-05-12

**Authors:** Sourav Maity, Monica Mazzolini, Manuel Arcangeletti, Alejandro Valbuena, Paolo Fabris, Marco Lazzarino, Vincent Torre

**Affiliations:** 1 International School for Advanced Studies (SISSA) Neuroscience Area, via Bonomea 265, Trieste 34136, Italy; 2 CBM S.c.r.l., Area Science Park, Basovizza, Trieste 34149, Italy; 3 IOM-CNR, Area Science Park, Basovizza, Trieste 34149, Italy

## Abstract

Cyclic nucleotide-gated (CNG) channels are activated by binding of cyclic nucleotides. Although structural studies have identified the channel pore and selectivity filter, conformation changes associated with gating remain poorly understood. Here we combine single-molecule force spectroscopy (SMFS) with mutagenesis, bioinformatics and electrophysiology to study conformational changes associated with gating. By expressing functional channels with SMFS fingerprints in *Xenopus laevis* oocytes, we were able to investigate gating of CNGA1 in a physiological-like membrane. Force spectra determined that the S4 transmembrane domain is mechanically coupled to S5 in the open state, but S3 in the closed state. We also show there are multiple pathways for the unfolding of the transmembrane domains, probably caused by a different degree of α-helix folding. This approach demonstrates that CNG transmembrane domains have dynamic structure and establishes SMFS as a tool for probing conformational change in ion channels.

## Abstract (web summary)

Cyclic nucleotide gated channels are activated after binding cyclic nucleotides. Here, using single molecule force spectroscopy, the authors reveal that cyclic nucleotide binding causes conformational changes and tighter coupling of the S4 helix to the pore forming domain.

## Graphical abstract

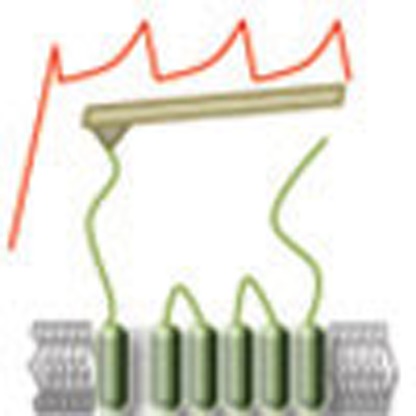

Atomic force microscopy (AFM) is a powerful technique used for surface imaging, measurements of sample mechanics and for the analysis of molecular interactions. Single-molecule force spectroscopy (SMFS) uses an atomic force microscope to apply a force to unfold a molecule or a polymer^1^,^2^,^3^,^4^. The obtained force–distance (*F–D*) curves characterize the stretching of the molecule; the resulting sequence of unfolding force peaks and their magnitude allows for the identification of folded and unfolded regions, thus providing insight into the interactions between and within domains of the molecule that stabilize secondary structures^1^,^2^,^3^,^4^,^5^. SMFS has been used to identify the conformational changes of membrane proteins belonging to the rhodopsin family^6^,^7^,^8^ and other proteins, such as the Na^+^/H^+^ antiporter, the BetP symporter, the KpOmpA transmembrane protein, the β2-adrenergic receptor, T4 lysozyme and the leucine-binding protein^9^,^10^,^11^,^12^,^13^,^14^.

Ion channels are membrane proteins that play a major functional role and they are grouped in superfamilies^15^,^16^: the superfamily of voltage-gated ion channels comprises Na^+^, K^+^ and Ca^2+^ channels, whose gating (transitions between the open and closed conformation) depends on the membrane voltage. This superfamily also includes cyclic nucleotide-gated (CNG) channels^17^,^18^,^19^,^20^,^21^ that are voltage dependent^21^ but are opened by the binding of cyclic nucleotides to the cyclic nucleotide-binding (CNB) domain^17^,^19^,^20^.

In vertebrates, seven members of the CNG channel gene family have been identified^19^,^22^ and are grouped into two subtypes, CNGA (CNGA1–CNGA5) and CNGB (CNGB1 and CNGB3). CNGA1, CNGA2, CNGA3 and CNGA5 (but not CNGA4) can form cyclic nucleotide-activated homotetrameric channels, while CNGB1 and CNGB3 are modulatory subunits that cannot form functional homomeric channels. Hydropathicity and biochemical analyses of CNGA1 channels^17^—690 amino acid residues (a.a.) long—have revealed six transmembrane α-helices (known as S1, S2, S3, S4, S5 and S6) that span the lipid bilayer (see Supplementary Fig. 1a); these helices are linked by non-spanning loops, which are either extracellular or intracellular. Ion permeation occurs through a pore region between S5 and S6, and electrophysiological experiments have identified 20 a.a. that form the P-helix (V348-L358) and the selectivity filter (T359-P367)^23^,^24^,^25^,^26^,^27^,^28^. The amino- and carboxy-terminal ends are both cytoplasmic, and the C-terminal end (N400-D690) is a large domain composed of the C-linker (N400-E482) and the CNB domain (A483-N610)^29^,^30^. The CNB domain shares 20% sequence identity with other CNB proteins, such as the CNB domain of HCN channels^31^ and MlotiK1 potassium channels (originally referred as mlCNG channels)^32^, and it consists of three α-helices and eight stranded anti-parallel β-rolls. The functional properties of CNG channels have been investigated extensively^19^,^33^,^34^, and a low-resolution architecture^35^, partial crystal structures of the CNB domain^31^,^36^,^37^, a crystal structure of the isolated C-terminal end from L621 to D690 (ref. 38) and a mimic of the pore^39^ are available. However, the full-length channel has never been crystallized and the conformational changes that are associated with gating are poorly understood.

In this study, we demonstrate how SMFS can be used to examine the gating of CNGA1 channels that are overexpressed in membranes from *X. laevis* oocytes^21^,^40^,^41^ (that is, almost *in situ*); the plasma membrane of these oocytes contains few native membrane proteins^41^,^42^,^43^,^44^. We identify *F*–*D* curves using bioinformatics analysis and by engineering proteins that are composed of CNGA1 channels linked at their C-termini to an SMFS marker, that is, a protein with a known unfolding pattern that act as a fingerprint. Our results provide new insights on the structure of the transmembrane domain of CNGA1 channels: first, the S4 domain shows different interactions between S3 and S5 in the closed and open state. Second, there are multiple pathways for the unfolding of the transmembrane domain probably caused by a different degree of folding of α-helices.

## Results

### CNGA1 channel constructs

Several constructs were designed to identify the *F*–*D* curves obtained from the unfolding of CNGA1 channels, to explore different pulling configurations and to validate a hypothesis of the molecular mechanisms. All these constructs had cGMP-activated currents that were measured using electrophysiological experiments (Supplementary Fig. 1 and Supplementary Note 1). We performed SMFS experiments in the presence and the absence of cGMP, that is, in the open and closed states of these channels. We performed SMFS experiments using both uninjected oocytes and oocytes injected with the messenger RNA coding for CNGA1 channels.

### SMFS of the CNGA1 channels

In our SMFS experiments, the obtained *F*–*D* curves could not only represent the unfolding of the full CNGA1 channel but also the unfolding of endogenous proteins and/or of the partial unfolding of CNGA1 channels. To identify the *F*–*D* curves obtained from the unfolding of the full CNGA1 channels, we have designed a method that analyses the *F*–*D* curves obtained using CNGA1 channels with appropriate mutations and/or channels that bear specific fingerprints.

The N- and C- termini and some loops between the transmembrane helices of the CNGA1 channels are cytoplasmic, and the AFM tip could attach to all a.a. in these different positions. If the tip starts the unfolding from residue D690 (that is, from the C-terminal)—assuming that the length of a single residue is 0.4 nm (ref. 1)—the complete stretch corresponds to a contour length (Lc) of ∼240 nm (from the end of the C-terminal to the beginning of S1); if the AFM tip started the unfolding from residue M1 (that is, from the N-terminal), the complete stretch corresponds to an Lc of 180 nm (from the N-terminal to the end of S6). Therefore, we have restricted our analysis to those *F*–*D* curves that had the last peak with an Lc value larger than 220 nm and only ∼1% of the *F*–*D* curves passed this filtering step (see Methods and Supplementary Fig. 2). These *F*–*D* curves were too diverse to be ascribed to the unfolding of the same protein. To identify the *F*–*D* curves obtained from the unfolding of CNGA1 channels, we developed a two-step method based on the following criteria: first, these *F*–*D* curves must be distinguishable and must be found only in SMFS experiments performed using membranes extracted from injected oocytes expressing CNGA1 channels at a high level; these *F*–*D* curves must stand out from the other *F*–*D* curves and must form a cluster of *F*–*D* curves with similar features (identification). Second, these *F*–*D* curves are ‘good’ candidates as *F*–*D* curves from the unfolding of CNGA1 channels, but they must be further validated by an appropriate fingerprint that is clearly visible in the *F*–*D* curves (validation).

**Figure 1:**
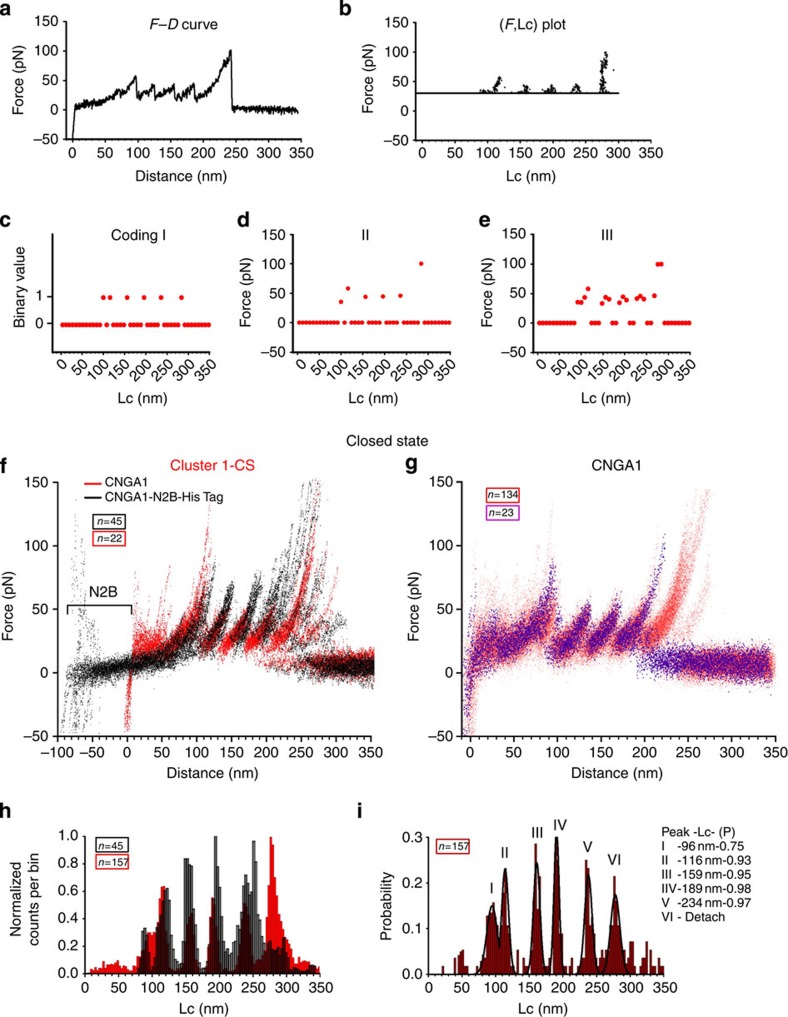
*F*–*D* curves from CNGA1 and CNGA1-N2B-HisTag in the closed state. (**a**) An example of an *F*–*D* curve. (**b**) Transformation of the *F*–*D* curve in **a** into an (*F*,Lc) plot; if the *F*–*D* curve fits well in a piecewise manner using the WLC model, the resulting (*F*,Lc) plot is composed of a series of almost vertical segments located at the corresponding value of Lc and the upper value of each of these segments corresponds to the value of the force peak F. (**c**–**e**) Three different coding schemes of increasing complexity that are all based on the processing of the (*F*,Lc) plot in **b**; coding scheme I (**c**) considers only the location of the force peak (the corresponding value of Lc) and is a binary coding; coding II (**d**) considers the location and the amplitude of the force peak and is not a binary coding; coding III (**e**) fits all sample values of *F* above 30 pN. Red dots represent the final coding of the *F*–*D* curves. (**f**) 22 *F*–*D* curves from Cluster 1-CS of CNGA1 (red) detected using bioinformatics analysis and 45 *F*–*D* curves from the construct CNGA1-N2B-HisTag (black) in the closed state. The construct with N2B has an initial flat region of 85 nm, followed by force peaks matching the unfolding events observed with the CNGA1 construct. (**g**) Superimposition of 157 *F*–*D* curves obtained from injected oocytes showing the peak force location of CNGA1. The 23*F*–*D* curves (violet) end with a force peak with an Lc of ∼234 nm; 134 *F*–*D* curves (red) have an additional force peak with an Lc of ∼276 nm. (**h**) Superimposition of histograms of normalized counts/bin against Lc from the 157 *F*–*D* curves of **b** (now all in red) and 45 *F*–*D* curves from the CNGA1-N2B-HisTag construct (black). (**i**) Histogram of Lc values of force peak (with Gaussian fit for the different peaks) from the *F*–*D* curves in **g** with five peaks located at 96±3 (mean±s.d., *n*=117), 116±3 (mean±s.d., *n*=146), 159±3 (mean±s.d., *n*=157), 189±5 (mean±s.d., *n*=157), 234±6 (mean±s.d., *n*=157) nm and the detachment with the probability (*P*) of 0.75, 0.93, 0.95, 0.98 and 0.97, respectively.

The bioinformatics analysis (see Methods) was based on the coding of the *F*–*D* curves (Fig. 1a). From the analysis of the corresponding (*F*,Lc) plot (Fig. 1b)^45^, three different coding schemes were obtained (Fig. 1c–e). Once the *F*–*D* curves were coded in appropriate strings of symbols, clustering methods developed in Computer Science were used (see Methods and Supplementary Figs 3 and 4). At the end of the bioinformatics analysis, we identified three major clusters of similar *F*–*D* curves that were obtained only from the membranes extracted from injected oocytes (Supplementary Fig. 5). To validate these clusters as clusters obtained from the unfolding of CNGA1 channels in the closed state, we used the construct CNGA1-N2B-HisTag and we selected those *F*–*D* curves that exhibited an N2B fingerprint (black curves in Fig. 1f) that showed an initial segment of ∼85 nm (in Lc) without unfolding events, which are typical characteristics of the N2B construct^46^,^47^,^48^. This initial segment was followed by an additional segment resembling the peaks obtained for the CNGA1 channel construct (red curves in Fig. 1f). *F*–*D* curves, obtained from the unfolding of CNGA1 channels, were identified by an initial filtering (Step 1 and 2 described in the Methods) of *F*–*D* curves from experiments using injected and uninjected oocytes followed by a clusterization aimed to identify clusters of *F*–*D* curves obtained only from injected oocytes (Step 3 and 4 in the Methods).

When these curves were displaced by Lc of 85 nm, the resulting curves superimposed precisely with the curves present in the Cluster 1-CS (Fig. 1f and Supplementary Fig. 5a). This cluster was identified using the bioinformatics analysis and was therefore validated as representing the *F*–*D* curves obtained from the C-terminal unfolding of the CNGA1 channels. These last *F*–*D* curves were used as a template to find additional *F*–*D* curves (Fig. 1g), which when shifted by <±5 nm aligned properly with those of Fig. 1f and include also *F*–*D* curves from Cluster 2-CS (Supplementary Fig. 5b) and additional *F*–*D* curves from injected oocytes (see Methods).

Some *F*–*D* curves (violet curves in Fig. 1g) ended with a force peak that had an Lc of ∼234 nm, whereas the remaining *F*–*D* curves (red curves in Fig. 1g) were longer and had an additional force peak with an Lc of ∼276 nm, which exactly correspond to 690 a.a. This behaviour is attributed to the variability of the final detachment. We computed the corresponding value of Lc (see Methods) for each point of the *F*–*D* curves, to compare the Lc histograms for the selected curves from the CNGA1 channel (Fig. 1h in red) and for the curves from the construct CNGA1-N2B-HisTag (Fig. 1h in black). When the Lc histogram that was obtained from the construct CNGA1-N2B-HisTag is shifted by 85 nm (the closeness between the two sets of *F*–*D* curves was quantified using the inter-cluster similarity described in Methods), the histogram of Lc values at force peaks from the CNGA1 channels has five common peaks (Fig. 1i).

**Figure 2:**
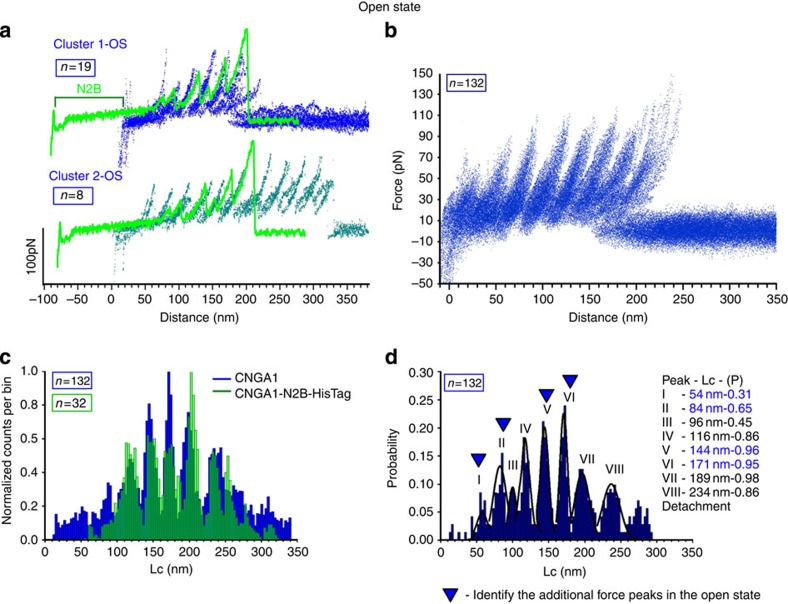
Unfolding of CNGA1 and CNGA1-N2B-HisTag in the open state. (**a**) Example of *F*–*D* curves obtained from the unfolding of single CNGA1 (blue and cyan) and CNGA1-N2B-HisTag (green) constructs in the open state (19 curves for cluster 1-OS, 8 curves for cluster 2-OS and 1 curve for CNGA1-N2B-HisTag). The construct with N2B has an initial flat region of 85 nm, followed by peaks that correspond to the unfolding events that were observed in the CNGA1 construct. (**b**) Superimposition of 132 *F*–*D* curves that were obtained from injected oocytes in the open state using the curves of Cluster 1-OS as a template. (**c**) Superimposition of the histograms of normalized counts/bin against Lc from the *F*–*D* curves of **b** (blue) and 32 *F*–*D* curves from the CNGA1-N2B-HisTag (green) constructs in the open state. (**d**) Histogram of Lc values of force peak (with Gaussian fit for the different peaks) from the *F*–*D* curves in **d** with eight peaks located at 54±3 (mean±s.d., *n*=41), 84±3 (mean±s.d., *n*=86), 96±3 (mean±s.d., *n*=59), 116±3 (mean±s.d., *n*=114), 144±3 (mean±s.d., *n*=132), 171±3 (mean±s.d., *n*=132), 189±5 (mean±s.d., *n*=132), 234±6 (mean±s.d., *n*=132) nm and the detachment; the probabilities of the unfolding are 0.31, 0.65, 0.45, 0.86, 0.96, 0.95, 0.98 and 0.86, respectively.

We performed SMFS in the open state (Fig. 2), to determine the conformational changes that occur upon gating^19^,^33^,^34^. Clustering procedures identified two major groups of *F*–*D* curves that were found only from membranes extracted from injected oocytes. The last force peak of the first cluster (Cluster 1-OS) had an Lc of <300 nm (blue curves in Fig. 2a), which was similar to that for Cluster 1-CS (Fig. 1f). However, we found another cluster (Cluster 2-OS) of *F*–*D* curves in which the last force peak had an Lc that was >350 nm (cyan curves in Fig. 2a); these curves appeared to be the sequential unfolding of the longer protein. The use of the N2B fingerprint (green curves in Fig. 2a) validated that Cluster 1-OS resulted from the unfolding of a single CNGA1 channel and suggested that Cluster 2-OS was obtained from the unfolding of two CNGA1 channel subunits that interact via the N-terminal of one subunit and the C-terminal of its neighbour^49^,^50^. The Lc histogram from the *F*–*D* curves of these two clusters superimpose very precisely up to 250 nm (Supplementary Fig. 6a). We found other *F*–*D* curves that could be ascribed to the unfolding of CNGA1 channels in the open state (Fig. 2b) when we used Cluster 1-OS as the template. Lc histograms that were obtained in both the closed and open states from the construct CNGA1-N2B-HisTag, and similar histograms that were shifted by 85 nm and obtained from the CNGA1 channels have common peaks (Fig. 2c). The histogram of the Lc that was obtained from the CNGA1 *F*–*D* curves in the open state has eight peaks (Fig. 2d) and was noticeably different from those obtained for the closed state (Figs 1i and 2d, and Supplementary Fig. 6b): first, there is a force peak with an Lc of 84±3 nm (mean±s.d., *n*=85) and a mean force of 60±15 pN (mean±s.d., *n*=85), which was rarely observed in the Lc histogram that was obtained from the closed state channel. Second, the force peak with an Lc of 159±3 nm (50±12 pN) (mean±s.d., *n*=149) that was observed from the closed state channel is replaced by two force peaks with Lc values of 144±3 nm (mean force of 65±22 pN) (mean±s.d., *n*=126) and 171±3 nm (mean force of 80±18 pN) (mean±s.d., *n*=126). All the other force peaks that are present in the closed state are also observed in the open state (Supplementary Figs 6b and 7).

For a further validation of the identification of *F*–*D* curves of Figs 1 and 2 as obtained from the unfolding of CNGA1 channels, we performed SMFS on the CNGA1–CNGA1 tandem^51^ construct (Supplementary Fig. 8 and Supplementary Note 3) and the F380C construct^52^ (Supplementary Fig. 9 and Supplementary Note 4). The results of the experiments with the CNGA1–CNGA1 tandem show that in the open state CNGA1 channels can be unfolded as a sequence of two linked subunits. The unique repetitive unfolding pattern of the *F*–*D* curves obtained from identical experiments also confirms that the *F*–*D* curves of Figs 1 and 2 represent the unfolding of the CNGA1 channel from the C-terminal end in the closed and open states, respectively. In the open state, the mutant channel F380C is known to form a disulfide bond between the exogenous C380 and the endogenous C314 (ref. 52), and the *F*–*D* curves obtained from its unfolding are expected to be 26.4 nm shorter than those obtained from the CNGA1 channels, in agreement with the experimentally observed gap of 26±2 nm (mean±s.d., *n*=28; Supplementary Fig. 9).

The comparison of the *F*–*D* curves of Figs 1 and 2 also demonstrates that the unfolding pathway of CNGA1 channels is different in the open and closed states, and that the unfolding of CNGA1 channels in the open state is characterized by the presence of three additional peaks.

### Identification of the cytoplasmic domain

**Figure 3:**
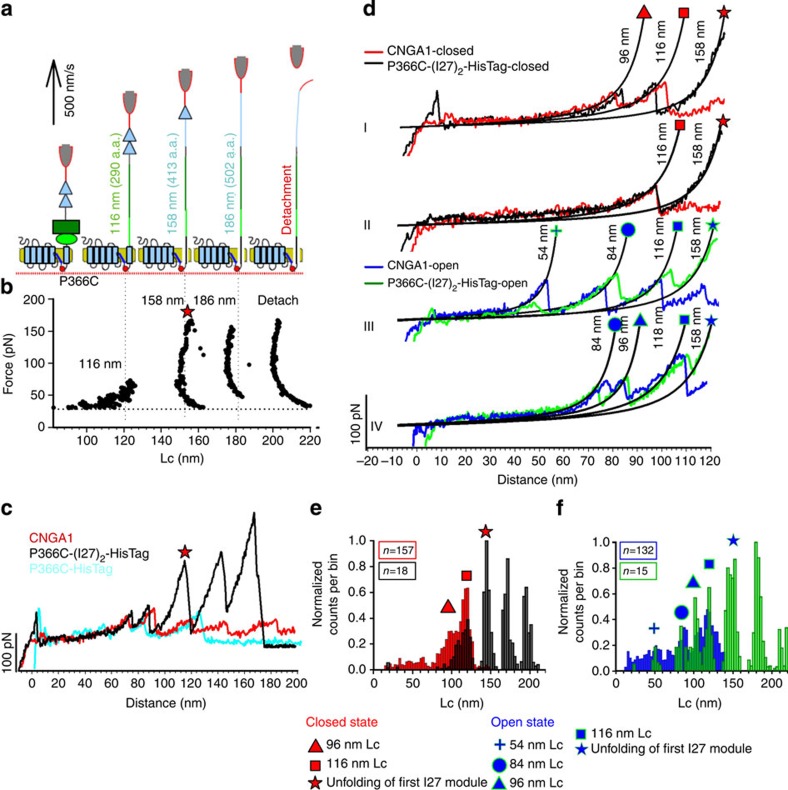
Unfolding of CNB domain. (**a**) Schematic representation of the unfolding sequence, with the expected Lc values and the relative a.a. length in parenthesis and single module of I27 (cyan triangle), CNB domain (green rectangle), C-linker (green oval), transmembrane domains (cyan rectangles) and mutation P366C (red dots). (**b**) (*F*,Lc) plot from a typical curve of P366C-(I27)_2_-HisTag. The finger print is composed by two force peaks of ∼200 pN separated by ∼28 nm with the detachment peak, with a value of Lc corresponding to unfolding of the cytoplasmic domain from D690 to P366 and the two I27 modules (that is, (D690−P366)·0.4 nm+2·28 nm))=186 nm. (**c**) Superimposition of the *F*–*D* curves of CNGA1 (red), P366C-HisTag (cyan) and P366C-(I27)_2_-HisTag (black). The *F*–*D* curves from the construct P366C-HisTag are shorter than those from CNGA1 and have a detachment event at 120–140 nm. Force peaks with Lc values at 96 and 116 nm are present in the *F*–*D* curves from the CNGA1, P366C-HisTag and P366C-(I27)_2_-HisTag constructs. (**d**) Different unfolding pathways in the open and closed states; I and II: superposition of the *F*–*D* curves from CNGA1 channels (red) and from the construct P366C-(I27)_2_-HisTag (black) in the closed state. In I, there are two force peaks with Lc values of 96 and 116 nm, but there is only one force peak in II; III and IV: superposition of the *F*–*D* curves from CNGA1 channels (blue) and from the constructs P366C-(I27)_2_-HisTag (green) in the open state. In III there are three force peaks with Lc values of 54, 84 and 116 nm, and in IV there are three force peaks with Lc values of 84, 96 and 118 nm. The force peak with an Lc of 158 is only present in the construct P366C-(I27)_2_-HisTag. (**e**) Superimposition of histograms of normalized counts/bin against Lc from the *F*–*D* curves obtained from P366C-(I27)_2_-HisTag (black) and from CNGA1 channels (red) in the closed state (**f**) same as in **e** for P366C-(I27)_2_-HisTag (green) and from CNGA1 channels (blue) in the open state. The data in **e**,**f** are from CNGA1 channels as described in Figs 1 and 2.

The Lc histograms in the closed (Fig. 1) and open (Fig. 2) states show force peaks with values of Lc ranging from 50 to 250 nm. It is important to identify the part of the *F*–*D* curves that corresponds to the unfolding of the cytoplasmic and transmembrane domains. This question can be answered by having *F*–*D* curves that represent the unfolding of only the cytoplasmic domain of CNGA1 channels in which the extracellular loop of S6 is strongly anchored to the substrate and the other portion of the transmembrane domain cannot be unfolded (Fig. 3a,b). Therefore, we constructed the mutant channel P366C-HisTag, in which we inserted a cysteine at position P366 on the extracellular loop of S6 (ref. 28). If this exogenous cysteine forms a covalent bond with a gold substrate—with a breaking force of 1.4 nN (ref. 53)—the transmembrane domain cannot be unfolded, and only the region from D690 to P366 will be unfolded, corresponding to ∼130 nm (Fig. 3c). To identify these shorter *F*–*D* curves, we inserted a fingerprint at the C-terminal end of the mutant channel P366C; this finger was composed of two I27 modules^1^,^46^,^54^ and we performed SMFS experiments with the construct P366C-(I27)_2_-HisTag. If the cantilever tip attaches to the HisTag and because CNGA1 unfolds with forces below 200 pN, we expect^4^ to unfold initially the cytoplasmic domain and S6 from D690 up to P366 and then the two I27 modules (Fig. 3a,b).

We found that many of *F*–*D* curves obtained from membranes extracted from oocytes injected with the construct P366C-(I27)_2_-HisTag exhibited the expected fingerprint (Fig. 3b and black curve in Fig. 3c). In the closed state, we observed a force peak with an Lc of 96±3 nm (mean±s.d., *n*=117; curve I in Fig. 3d). In the open state, we observed two additional force peaks with Lc values of 54±3 (mean±s.d., *n*=41) and 84±3 nm (mean±s.d., *n*=86; curves III and IV, respectively, in Fig. 3d). All these force peaks were observed in CNGA1 channels (red and blue curves in Fig. 3d) and in the construct P366C-(I27)_2_-HisTag (black and green curves in Fig. 3d). In the closed state, the *F*–*D* curves from the construct P366C-HisTag (cyan curve in Fig. 3c) had two peaks with Lc values of 96±3 (mean±s.d., *n*=18) and 116±3 nm (mean±s.d., *n*=18), followed by a detachment. For both constructs, these features were preceded by a less frequent and lower force peak with an Lc that varied between 80 and 100 nm (Fig. 3e). When the same experiments were repeated in the open state, the force peaks had similar values for both the CNGA1 channel and the construct P366C-(I27)_2_-HisTag (Fig. 3f). The *F*–*D* curves for the P366C-(I27)_2_-HisTag and CNGA1 constructs can be closely superimposed for the portions of the curve up to the force peaks with Lc values of 116±3 nm in both the closed and open states, which indicates that the cytoplasmic domain of the CNGA1 channels unfolds before the transmembrane segments S1–S6. These results show that the portion of the *F*–*D* curves with tip-sample separation (TSS) values <116 nm (corresponding to ∼290 a.a.) represents the unfolding of the cytoplasmic domain comprising both the CNB domain and the C-linker (from D690 to N400). The assignment of force peaks, in this region of the *F*–*D* curves, to the unfolding of specific molecular domains is more difficult.

### Conformational changes upon gating in the cytoplasmic domain

**Figure 4:**
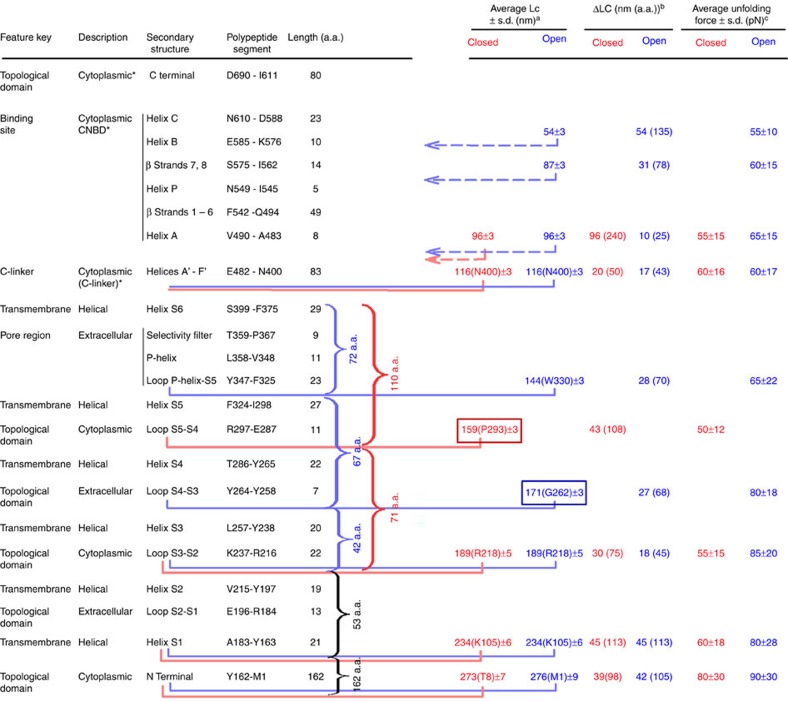
Assignment of secondary structure and different domains of CNGA1 channels to the observed force peaks in the closed and open states. The first and second columns show the key features of the functional domains identified in CNGA1 channels according to the available literature^23^,^24^,^25^,^26^,^27^,^28^,^29^,^30^; the third column reports the presumed secondary structure^17^,^31^; the fourth and fifth columns contain the corresponding polypeptide segments and the associated a.a. number; the remaining columns indicate the Lc, ΔLc and force values of the corresponding force peaks in the closed and open states. The length and the polypeptide segments are obtained from the results of Figs 1 and 2. The number of a.a. for the transmembrane domains has been calculated by considering the membrane thickness (∼5 nm or ∼13 a.a.)^3^,^44^. The initial and final a.a. of each polypeptide segment are only indicative (**a**) average Lc values of the force peaks with s.d. in nanometres, with the number inside the brackets representing the average position of the force peaks in the a.a. sequence. (**b**) Average ΔLc in nanometres and the corresponding number of a.a. (in brackets). (**c**) Average value of the unfolding force with the corresponding s.d. in pN. *The unfolding of the cytoplasmic segments in the C-terminal cannot be exactly determined.

Our results indicate three conformational changes that occur in three regions upon gating in CNGA1 channels: the cytoplasmic domain, the transmembrane domain and at the C and N termini. The assignment of the secondary structure of CNGA1 channels corresponding to the observed force peaks in the closed and open states (Figs 1 and 2) is reported in Fig. 4.

The unfolding of the cytoplasmic segment up to an Lc of 80 nm in the closed state usually requires forces below 25–35 pN, consistent with the notion that these domains do not have a well-defined conformation^30^ and with their problematic crystallization^31^,^35^,^36^,^37^. In the open state, there is a peak, which appears with a probability of ∼0.31, that has an Lc of 54±3 nm (mean±s.d., *n*=41) with a force of 55±10 pN (mean±s.d., *n*=41); this peak is either absent or present with a very low force in the closed state. The force peak with an Lc of 84±3 nm (mean±s.d., *n*=86), a force of 60±15 pN (mean±s.d., *n*=86) and probability of 0.65 is only present in the open state, whereas the force peak with an Lc of 96±3 nm (mean±s.d., *n*=117) is present both in the open and closed states with a probability of 0.45 and 0.75, respectively.

### Conformational changes upon gating in the membrane domain

**Figure 5:**
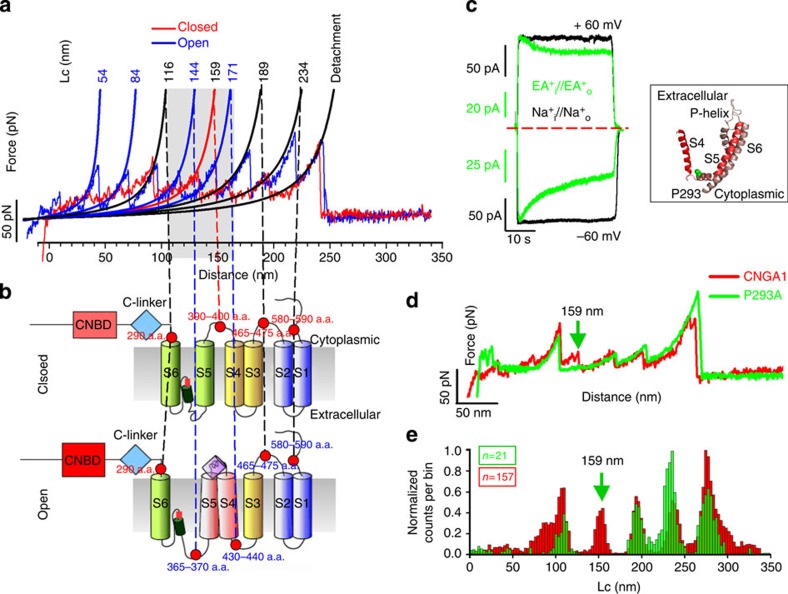
Conformational changes in the transmembrane domain during the gating of CNGA1 channels. (**a**) Superposition of two representative *F*–*D* curves of CNGA1 channels in the closed (red) and open (blue) states. Continuous black lines obtained from the fitting with WLC model. Numbers indicate the corresponding values of Lc. (**b**) Schematic representation of hypothesized interactions between the transmembrane helices in the closed and open states. Red dots indicate the approximate location of the force peaks and the numbers of the corresponding a.a. (**c**) Electrophysiological recordings from mutant channel P293A in the presence of Na^+^ (black) and ethylammonium (EA^+^) (green) at ±60 mV. In the black box, the homology model of the S4 and S6 transmembrane domains of the CNGA1 channel based on the molecular structure of the Kv1.2 channel; the conserved P293 is indicated in green. (**d**) Superposition of two representative *F*–*D* curves for the CNGA1 channel (red) and for the P293A (green), both in the closed state. The green arrow, corresponding to the value of Lc=159 nm, indicates the differences between the two constructs. (**e**) Superimposition of histograms of normalized counts/bin against Lc from the 157 *F*–*D* curves of CNGA1 (red, the same as in Fig. 1h) and 21 *F*–*D* curves from mutant channel P293A in the closed state (green), both in the closed state. Arrow as in **d**.

The force peaks with an Lc between 120 and 250 nm appear with a probability close to 1 and correspond to the unfolding of the transmembrane domains that unfold sequentially according to the a.a. sequence of the protein^3^,^55^, with force peaks corresponding to unstructured regions, such as the connecting loops^11^; these force peaks can be reliably assigned to specific domains of CNGA1 channels (Fig. 5a,b).

In the closed state (Fig. 5a,b), three peaks with Lc equal to 159±3 (mean±s.d., *n*=157), 189±3 (mean±s.d., *n*=157) and 234±3 nm (mean±s.d., *n*=157) are observed requiring forces varying between 50 and 60 pN (Fig. 1 and Supplementary Fig. 7). The peak at 159±3 nm (mean±s.d., *n*=157) corresponds to the unfolding of S6-P-helix-S5 segments (S399-L301) followed by an unstructured loop (N300-N291) that causes a drop in the pulling force; the second peak at 189±3 nm (mean±s.d., *n*=157) corresponds to the unfolding of S4 and S3 (N291-V215), and the last peak at 234±3 nm (mean±s.d., *n*=157)—before detachment—corresponds to the unfolding of S2 and S1 (R216-E100). The force peak that has an Lc of 159±3 nm (mean±s.d., *n*=157) for the closed state (corresponding to a residue near P293) splits into two force peaks for the open state with Lc of 144±3 (mean±s.d., *n*=132) (∼360 a.a.) and 171±3 (mean±s.d., *n*=132) (∼428 a.a.) nm; these peaks have higher forces of 65±22 (mean±s.d., *n*=132) and 80±18 pN (mean±s.d., *n*=132), respectively (Figs 2 and 5a, and Supplementary Fig. 7). This splitting reveals an important conformational change in the transmembrane domain that could be controlled by the S4–S5 linker via the helix-breaker proline P293: in the open state, the unfolding of S6-P-helix-S5 occurs in two steps. The first step consists of the unfolding of S6 and the P-helix (V348-S399); this unfolding is followed by a drop in the force at an unstructured loop (F325-Y347). In the second step, S5 is unfolded together with S4 (Y265-F324). Therefore, in the open state S5 is mechanically coupled to S4, whereas in the closed state S5 is more strongly connected to the P-helix and S6, as suggested by the obtained *F*–*D* curves (Fig. 5b).

Homology modelling of the structure of CNGA1 channels based on the Kv1.2 channel^56^,^57^ and the use of an improved algorithm for predicting α-helix folding suggest that the stretch of a.a. from Y265 to F324, corresponding to the region comprising the S4 and S5 transmembrane domains, has a good propensity to have an α-helix fold and the three-dimensional (3D) structure is shown in Fig. 5c (inset). To validate this interpretation of the SMFS data (Figs 4 and 5a,b), we performed electrophysiological experiments using CNGA1 mutant channels with the following rationale: if S4 in the open state is mechanically coupled by the S4–S5 linker to S5 (Fig. 5c), point mutations in the S4–S5 linker are expected to propagate to the pore region and affect ionic permeation in the open state. We have identified a specific residue in the S4–S5 linker that corresponds to a conserved proline (P293 in the CNGA1 channel), which—when inserted into an α-helix—is expected to modify the ideal helical structure; therefore, we constructed the mutant channel P293A, in which P293 is substituted by the α-helix-forming alanine. It is well known that CNGA1 channels do not inactivate^21^, that is, no time-dependent inactivation in the cGMP-activated current is observed, neither in the presence of small permeant cations such as Na^+^ nor in the presence of larger organic cations such as ethylammonium (EA^+^). In the mutant channel P293A (Fig. 5c), the cGMP-activated current inactivated depending on the voltage in the presence of EA^+^ (green traces); however, in the presence of Na^+^ (black traces) there was no inactivation of the current both at positive and negative voltage, indicating that mutations in the S4–S5 linker affect ionic permeation and therefore, validating the notion that in the open state, the S4–S5 linker is mechanically coupled to the pore region. To better analyse the role of this proline, we performed SMFS experiments with the mutant channel P293A as well (Fig. 5d,e). In the closed state, the force peak with Lc around 159 nm is not present in the majority (about 80%) of *F*–*D* curves as evident from the comparison of *F*–*D* curves (Fig. 5d) and the histograms (Fig. 5e) of the CNGA1 channels (red) and mutant channels P293A (green). The absence of the force peak at 159 nm in the mutant channel P293A strongly suggests that S6, S5, S4 and S3 in this mutant channel, in the closed state, are mechanically coupled and unfold together, validating the notion that P293 is an α-helix breaker.

The determination of the exact value of Lc for a given force peak is limited by the shift (<±5 nm) used to align different *F*–*D* curves (Figs 1 and 2). This limitation is circumvented when the increase in contour length (ΔLc) is measured. ΔLc is a structural parameter determined by the number of a.a. residues involved in the folded structure^58^, providing information on the kinetic barriers for the unfolding as well^10^. We have analysed the variability of the ΔLc between the force peaks with Lc values around 159–189 nm, corresponding to the unfolding of S4 and S3 in the closed state (ΔLc_closed(3/4)_) and the variability of the ΔLc between the force peaks with Lc values around 171–189 nm, corresponding to the unfolding of S3 in the open state (ΔLc_open(3)_). We have also computed the sum of ΔLc corresponding to the unfolding of all the transmembrane domains from S6 to S1 in the closed (ΣΔLc_closed(TM)_) and open (ΣΔLc_open(TM)_) state.

**Figure 6:**
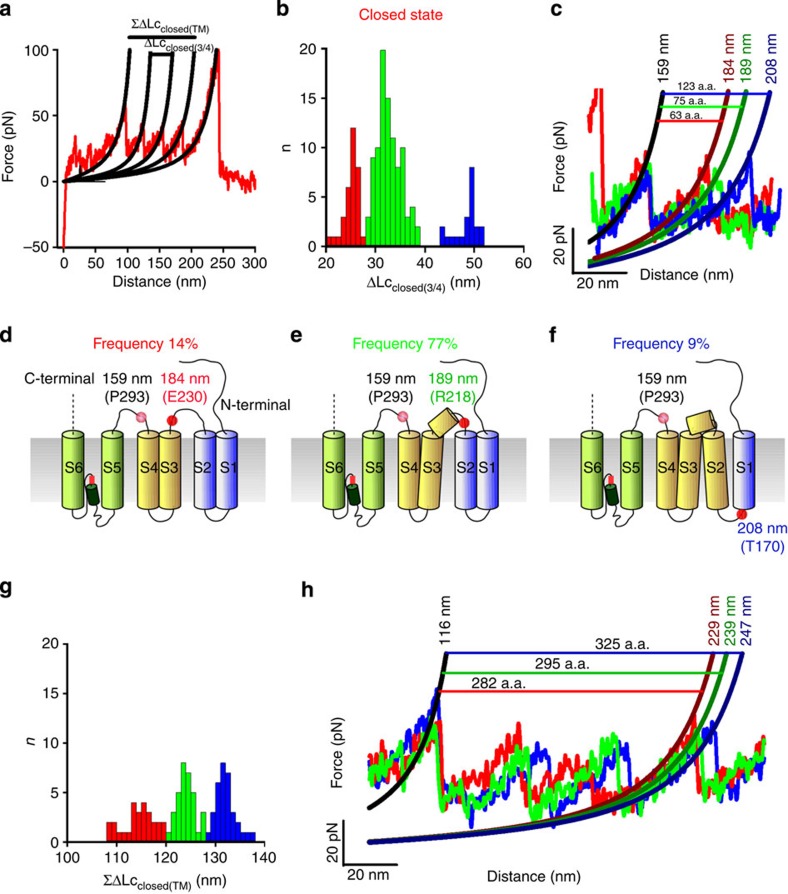
Variability of the unfolding of the transmembrane segments in the closed state. (**a**) A representative *F*–*D* curve in the closed state (the same as in Fig. 5a). Continuous black lines obtained from the fitting with WLC model. Horizontal lines indicate the corresponding values of ΔLc and ΣΔLc. (**b**) Histogram of ΔLc_closed(3/4)_ (bin 2 nm) in the closed state between the force peaks with Lc values around 159 to 189 nm. Colours correspond to different clusters of *F*–*D* curves with values of ΔLc_closed(3/4)_ around 26, 34 and 50 nm. (**c**) Superposition of representative *F*–*D* curves of the three clusters of **b** and the relative number of stretched a.a. (**d**–**f**) Schematic representation of the corresponding unfolding pathways. Small red dots or circles indicate the position of the force peaks, and the number is the corresponding value of Lc. The a.a. that presumably corresponds to the force peak is also indicated. (**g**) ΣΔLc_closed(TM)_ histogram (bin 1 nm) in the closed state for the force peaks as in **a** with values around 113, 123 and 131 nm. (**h**) Superposition of representative *F*–*D* curves of the three clusters of **g** and the relative number of stretched a.a.

**Figure 7:**
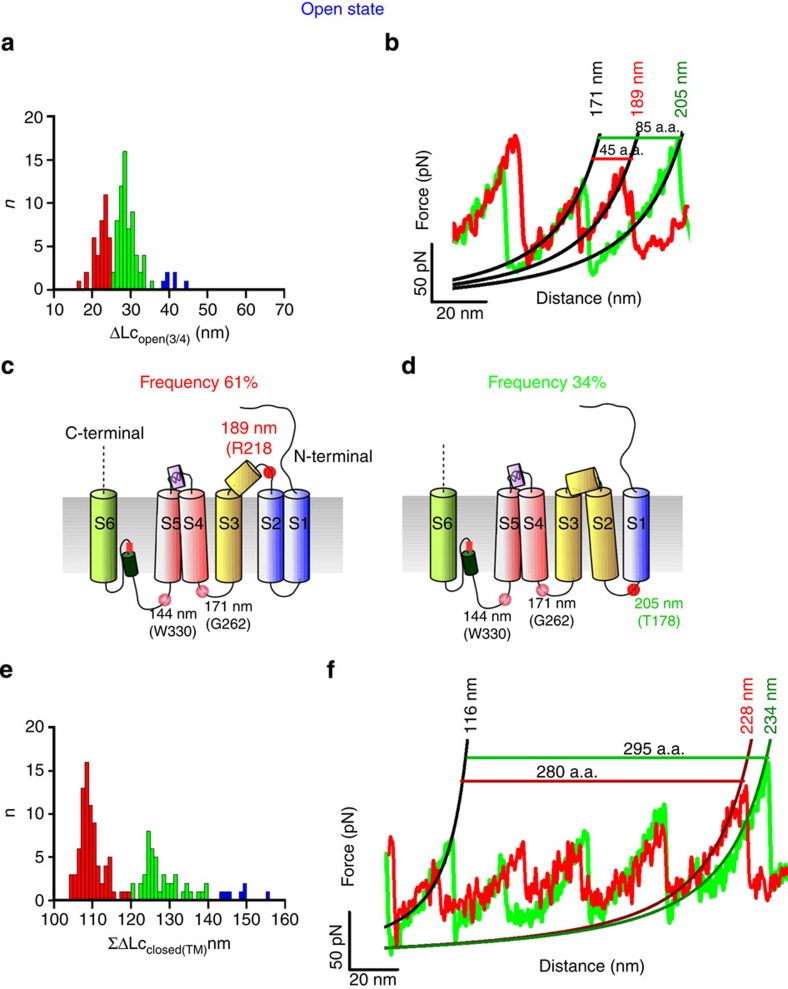
Variability of the unfolding of the transmembrane segments in the open state. (**a**) Histogram of ΔLc_open(3/4)_ (bin 2 nm) in the open state. (**b**) Superposition of representative *F*–*D* curves of the two clusters of **a** and the related number of stretched a.a. (**c**,**d**) Schematic representation of the corresponding unfolding pathways. Small red dots or circles indicate the position of the force peaks and the number is the corresponding value of Lc. The a.a. that presumably corresponds to the force peak is also indicated. (**e**) ΣΔLc_open(TM)_ histogram (bin 1 nm) in the open state. (**f**) Superposition of representative *F*–*D* curves of the two clusters of **e** and the related number of stretched a.a.

The distribution of ΔLc_closed(3/4)_ has three distinct peaks, corresponding to the stretching of a different number of a.a. (Fig. 6a–c) that have a similar unfolding force (that is, same mechanical stability). The unfolding with a ΔLc of 26 nm occurs with a low probability (14%) and corresponds to the unfolding of S3 and S4 from approximately P293 up to E230 (Fig. 5d). The most probable (77%) unfolding of S3 and S4 occurs with a longer ΔLc of 34 nm from approximately P293 up to R218. In this case, residues from E230 to R218 are mechanically coupled to S3, possibly being part of the α-helix-forming S3 (Fig. 6e). The unfolding with a ΔLc of 50 nm occurs with a lowest probability (9%) and corresponds presumably to the unfolding of S2 and S4 from approximately P293 up to T170 (Fig. 6f). In this case, S2 appears to be mechanically coupled to S4 and S3, suggesting that the connecting loop between S2 and S3 acts as a rigid handle and possibly forms a short α-helix. The distribution of ΣΔLc_closed(TM)_ has several peaks at about 113, 123 and 131 nm (Fig. 6g,h), indicating a significant variability of the unfolding of the entire transmembrane domain. This variability is likely to originate from the unfolding of folded and partially folded α-helices forming the transmembrane domain^58^. Similar results were obtained when we analysed *F*–*D* curves obtained in the open state (Fig. 7) of CNGA1 channels. The distribution of ΔLc_open(3/4)_ has distinct peaks, corresponding to the stretching of a different number of a.a. (Fig. 7a,b) and the unfolding with the two different ΔLc (Fig. 7c,d) occurs with a probability of 61% and 34%, respectively. The distribution of ΣΔLc_open(TM)_ has two peaks (Fig. 7e,f), indicating a variability of the unfolding of the entire transmembrane domain. These results (Figs 5, 6, 7) show that the transmembrane α-helices S2, S3, S4 and S5 have a variable mechanical coupling, and that this coupling differs in the closed and open states.

In the open state the force necessary to unfold the transmembrane domain varies between 65 and 85 pN, whereas in the close state this force varies between 50 and 60 pN (Figs 1 and 2, and Supplementary Fig. 7). This difference in the unfolding force was observed when CNGA1 channels were fused with the N2B fingerprint and in the CNGA1–CNGA1 tandem construct. The force necessary to unfold the transmembrane segments of other membrane proteins increases upon the binding of the appropriate ligands^13^, and the observed increase in the unfolding force is comparable to the force observed in CNGA1 channels. In β2-adrenergic receptors, ligand binding is thought to change the conformational and mechanical properties of the transmembrane segments of the receptor itself^13^. The breaking of a single H-bond requires a force of ∼4 pN (refs 8, 59), whereas the breaking of a single hydrophobic bond requires ∼30 pN (refs 8, 53) and breaking a single noncovalent bond requires a larger force of ∼160 pN (refs 8, 53). Therefore, the observed increase in the force necessary to unfold the transmembrane domain in the open state can be accounted for by the establishment of some H-bonds and/or of one or two additional hydrophobic interactions. Another possibility is that in the open state, more residues are folded as α-helices, such as the residues in the loop connecting S4 and S5.

## Discussion

We have performed SMFS experiments to recover structural information about CNGA1 channels in a physiological-like environment, thus avoiding purification and reconstitution into lipid bilayers. *F*–*D* curves obtained from the unfolding of CNGA1 channels expressed in *X. laevis* oocytes were identified using bioinformatics analysis validated by the N2B and I27 fingerprints, and were corroborated by experiments with CNGA1–CNGA1 tandem constructs and with the mutant channel F380C. Presented results provide new insights on the function of CNGA1 channels and show that S4 in the closed state is mechanically coupled to S3, but in the open state is mechanically coupled to S5. P293 in the S4–S5 linker and G262 in the loop connecting S3 and S4 could be the hinges regulating the mechanical coupling between these α-helices.

SMFS experiments also reveal a significant variability in the unfolding of the transmembrane domains (Fig. 6). This variability can originate from the existence of multiple unfolding pathways^10^,^14^,^58^ and/or from the existence of different initial conformations^10^,^14^,^58^. The variability of ΣΔLc_(TM)_ (see Fig. 6) can be explained if the α-helices of the transmembrane domain can be properly folded but also partially folded, suggesting that these α-helices are highly dynamic and do not have a fixed mechanical stability and folding^10^,^14^,^58^.

SMFS experiments can detect changes in the interactions of proteins that are not necessarily associated with changes in the secondary structure^60^,^61^, and for a proper interpretation of the changes in the portion of the *F*–*D* curves (Fig. 3) corresponding to the cytoplasmic domain, we have used additional experimental insight from nuclear magnetic resonance and X-ray approaches (Fig. 4). The unfolding of the cytoplasmic segment up to an Lc of 80 nm in the closed state requires forces below 25–35 pN, consistent with the notion that these domains do not have a well-defined conformation^30^. If the cytoplasmic domain of CNG channels does not have a well-defined 3D structure, it will be difficult to crystallize^31^,^35^,^36^,^37^, in agreement with the recent AFM imaging of the CNB domain of the of MlotiK1 potassium channels, which has a tetrameric well-defined structure only in the presence of cyclic nucleotides^32^. The 3D structure of the CNB domain of HCN channels has been determined in the presence of cAMP by X-ray crystallography^31^,^37^ and in the absence of cyclic nucleotide by nuclear magnetic resonance spectroscopy^36^. The comparison of these structures shows that in the absence of cyclic nucleotides some α-helices are either not folded or only partially folded. Therefore, the force peak with an Lc of 54±3 nm (mean±s.d., *n*=41) (∼135 a.a.) present in the open state (Figs 2 and 5) with a force larger than that seen in the closed state could correspond to the unfolding of α-helices in the CNB domain of CNGA1 channels, possibly the C (23 a.a.) and B (10 a.a.) helices, not folded in the absence of cGMP^30^. The force peak seen only in the open state with an Lc of 84±3 nm (mean±s.d., *n*=86; Figs 2 and 4) could correspond to some other conformational change associated to tetramerization of the CNB domain^37^. However, these experiments do not allow us to distinguish between a tetramer and a dimer of dimers. The force peak at 116 nm (Figs 1, 2 and 5) corresponding to ∼290 a.a. corresponds to the complete unfolding of C-termini till the end of S6 (a.a. 400).

In the closed state, some *F*–*D* curves terminate with a force peak with an Lc of 234 nm, but other *F*–*D* curves have an additional force peak with an Lc of ∼276 nm (Fig. 1f), which exactly correspond to 690 a.a. (276 nm/0.4 nm=690 a.a., that is, the total length of CNGA1 channels). Nonetheless, all these *F*–*D* curves are very similar before the force peak with an Lc of ∼234 nm. If the N-terminal does not interact with the C-terminal of a neighbouring subunit or with other domains of the same subunit, detachment of the cantilever tip can occur simultaneously with the unfolding of S1; however, without N-terminal interactions, an additional pull is required for the final detachment. Some of the *F*–*D* curves obtained in the open state were longer (Fig. 2a,b) and the force peaks appeared to be replicated, suggesting that these curves do not correspond to the unfolding of a single CNGA1 subunit but instead represent the unfolding of two interacting subunits. Indeed, in the open state, the N-terminal of one subunit strongly interacts with the C-terminal of a neighbouring subunit^49^,^50^; thus, two neighbouring subunits are almost linked together. Under these conditions, when the cantilever tip unfolds one CNGA1 subunit, an additional subunit can subsequently be unfolded. Therefore, our results indicate a possible interaction between the N- and C-termini in the closed state and this interaction is clearly potentiated in the open state. In the open state, we never observed ‘duplicated’ *F*–*D* curves when pulling with the constructs CNGA1-N2B-HisTag, because in these constructs the added domains do not allow the N- and C-termini to interact as they do in the CNGA1 channels. The interaction between the C- and N-termini in the open state can also explain the lower success of SMFS at obtaining good and complete *F*–*D* curves (Fig. 5), because the cantilever tip has limited access to the C-terminal.

Which is the possible molecular mechanism underlying the gating of CNGA1 channels? The results presented here and previous experimental observations suggest a more accurate view of the molecular mechanism underlying gating. Upon the binding of cGMP to the CNB domain that is almost located at the C-terminal end of CNGA1 channels, several conformational changes occur in the cytoplasmic domain, the transmembrane domain, and the C- and N-termini. The structural information obtained in the present investigation and in similar ion channels, such as the K, HCN and MlotiK1 potassium channels^31^,^32^, provides a better picture of the gating in CNG channels. Upon the binding of cGMP, the entire cytoplasmic domain acquires a more structured conformation^30^, either as a dimer of dimers^30^,^35^,^62^ or as a tetramer^63^. Similar to the observations in MlotiK1 potassium channels^64^, the cytoplasmic domain of CNGA1 could move vertically towards the membrane and could induce rotations, vertical shifts and tilts of the S5 and S6 transmembrane domains^24^,^27^, leading to widening of the filter region, where the gate is located^26^,^28^.

The S4–S5 linker of CNGA1 channels is composed of 11 a.a., in which the α-helix breaker P293 is flanked on the right by predominantly hydrophobic residues and flanked on the left by predominantly hydrophilic residues^57^, and the segment TNYP has a low propensity to be in an α-helical conformation^56^. Electrophysiological experiments with chimeric channels show that the C-linker interacts with the S4–S5 linker^57^. Therefore, we propose that in the closed state the cytoplasmic end of S4 is unfolded, but in the open state it becomes an α-helix due to its interaction with the C-linker; this change increases the mechanical coupling between S5 and the voltage sensor in S4. As a consequence, CNGA1 channels acquire voltage gating in the open state, as recently shown when large cations such as Cs^+^, methylammonium and dimethylammonium are the permeant ions^21^. A similar interaction between the cytoplasmic domain and the S4–S5 linker has been observed in CNG, Kv1.2 and hERG channels^57^,^65^.

In summary, new insights on CNGA1 channels emerge from this analysis. The S4 domain is mechanically coupled to S3 in the closed state but to S5 in the open state (Fig. 5); moreover, there are multiple pathways for the unfolding of the transmembrane domain (Figs 6 and 7) and the degree of folding of α-helices forming the transmembrane domain varies (Fig. 6) possibly also assuming a 3_10_ helix conformation^66^,^67^. These experimental observations, obtained from SMFS, show that the transmembrane domain has a dynamical structure. Moreover, our results show that SMFS is a powerful tool for analysing ion channels, suggesting that the same approach combining SMFS with informatics, mutagenesis and fusion with known fingerprints can be used to study other ion channels and membrane proteins, and to detect their conformational changes at the single-molecule level and in a physiological-like environment.

## Methods

### Molecular biology and CNGA1 constructs

Eight different channel constructs (see Supplementary Fig. 1 and Supplementary Note 1) were cloned into the expression vector known as pGEM-HE^38^. The constructs are the CNGA1 channel cloned from bovine retinal rod photoreceptors, as previously described^17^, which consists of 690 a.a. (here named the CNGA1 channel), the CNGA1 channel with a tag that is composed of six histidines (HisTag) at the C-terminal (CNGA1-HisTag), the CNGA1 with an N2B module (CNGA1-N2B-HisTag) and the CNGA1 with two I27 modules followed by the same tag [CNGA1-(I27)_2_-HisTag]. The HisTag was used to increase the probability of a specific attachment between the protein and the functionalized cantilever tip, but because of the lower level of expression of the construct CNGA1-HisTag (Supplementary Fig. 1b), we used the construct CNGA1 without the HisTag. Nevertheless, the HisTag was used with the other constructs, because they had a higher level of expression (Supplementary Fig. 1). We used two fingerprints, the N2B module (the shortest titin isoform)^46^,^47^,^48^ and two I27 modules (Ig module 27 of the I band of titin)^46^,^54^, to differentiate the *F*–*D* curves that were obtained from the unfolding of the CNGA1 channels from those of native membrane proteins that were present in the oocyte plasma membrane. The N2B module is composed of 210 a.a. with the mechanical properties of a random coil unfolding that does not show any unfolding events^47^,^48^. The unfolding of the I27 modules (each 89 a.a. long) shows the characteristic sawtooth pattern, in which successive force peaks have an increase in contour length (ΔLc) of ∼28 nm and amplitudes of ∼200 pN. The CNGA1–CNGA1 tandem^51^ construct was obtained using two identical CNGA1 subunits linked by a short 10 a.a. linker (GSGGTELGST) between the C-terminal end of the first subunit and the N-terminal end of the second subunit. The construct was generated by the insertion of one copy of the DNA coding for CNGA1 into a vector pGEMHE already containing another copy of DNA coding for CNGA1. The second subunit was made by replacing the ApaI restriction site (GGGCCC) at the end of the CNGA1 without changing the a.a. (GGTCCC) and adding at the start codon a new ApaI restriction site followed by the linker, using a PCR reaction (Platinum Pfx DNA polymerase, Invitrogen). Subunits were linked after HindIII/ApaI cut using T4 DNA Ligase (Promega). We performed a point mutation in an exposed loop of the channel (P366C) (two constructs: P366C-HisTag and P366C-(I27)_2_-HisTag), which is able to bind the gold surfaces used in our experiments. We also used two additional mutant channels: F380C and P293A. Single-residue mutagenesis was performed as described using the Quick Change Site-Directed Mutagenesis kit (Stratagene). Point mutations and cloning were confirmed by sequencing, using the sequencer LI-COR (4,000 l). The constructs CNGA1-N2B-HisTag and CNGA1-(I27)_2_-HisTag were cloned by DNA2.0 (Menlo Park, CA) than subcloned in pGEMHE; in both cases the linker between the CNGA1 and the N2B or I27 was the same of the CNGA1–CNGA1 tandem and the restriction enzyme sites used for the cloning were HindIII/ApaI. Complementary DNAs were linearized with NheI and were transcribed to RNA *in vitro* using the mMessage mMachine kit (Ambion, Austin, TX). All the restriction enzyme were from BioLabs (New England). The sequences for oligonucleotide primers and the sequences for the construct with N2B and I27 were included in the Supplementary Table 1.

### Heterologous expression system and sample preparation

Purified RNA of the different constructs was injected into *X. laevis* oocytes (‘Xenopus express’ Ancienne Ecole de Vernassal, Le Bourg 43270, Vernassal, Haute Loire, France). Oocytes were prepared as described^30^. *X. laevis* frogs were anaesthetized by immersion in a 0.2% aqueous solution of MS-222 (tricaine methanesulfonate) for ∼20 min. The anaesthetized animal was put on its back on a box of ice and a small incision of about 1 cm was made with a scalpel, laterally on the abdomen. Once the skin and the underneath abdominal muscles were cut through, the ovarian lobes became visible. The follicle (oocytes and follicle cells) were surgically removed from the ovarian lobes and placed in a Barth solution containing the following (in mM): 88 NaCl, 1 KCl, 0.82 MgSO_4_, 0.33 Ca(NO_3_)_2_, 0.41 CaCl_2_, 2.4 NaHCO_3_ and 5 TRIS-HCl (pH 7.4 buffered with NaOH). Follicles were separated in small groups and incubated for 1 h at 18 °C in a Barth solution without calcium, but supplemented with 1 mg ml^−1^ of collagenase. After this treatment, the residual follicle were removed manually with forceps. Selected oocytes were injected and maintained at 18 °C in a Barth solution that was supplemented with 0.05 mg ml^−1^ of gentamycin sulfate. Cells were incubated for 2–6 days. The vitelline membrane of oocytes was removed mechanically before the experiments. These oocytes were incubated on a freshly cleaved mica sheet (or on a gold surface) for 1–10 min in Standard solution (in mM) (110 NaCl, 10 HEPES and 0.2 EDTA; pH 7.4 buffered with NaOH) with or without 2 mM cGMP. We removed the cytoplasmic content (yolk and granules) using five to ten washings with the Standard solution after the membrane had been attached to the surface. The usual salts and reagents were purchased from Sigma-Aldrich (St Louis, MO, USA).

### Electrophysiological recordings

The functionality of all constructs was verified by exposure to 2 mM cGMP (saturating concentration) and then recording the channel current using electrophysiological measurement in the excised patch configuration (see Supplementary Fig. 1a–g). cGMP-gated currents in a voltage-clamp condition were recorded using a patch-clamp amplifier (Axopatch 200, Axon Instruments Inc., Foster City, CA, USA) 2–6 days after RNA injection at room temperature (20–24 °C), using borosilicate glass pipettes with resistances of 2–5 MΩ. The perfusion system allowed a complete solution change in <1 s. During the experiments, oocytes were kept in Ringer’s solution containing the following (in mM): 110 NaCl, 2.5 KCl, 1 CaCl_2_, 1.6 MgCl_2_ and 10 HEPES-NaOH (pH 7.4 buffered with NaOH). The Standard solution on both sides of the membrane consisted of (in mM) 110 NaCl, 10 HEPES and 0.2 EDTA (pH 7.4 buffered with NaOH). We used Clampex 10.0, Clampfit 10.1 and SigmaPlot 9.0 for data acquisition and analysis. Data are usually given as the mean±s.e.m. We attempted SMFS only in oocytes in which the measured cGMP-activated current was larger than 1 nA at ±100 mV.

### AFM and cantilever functionalization

The NanoWizard 3 AFM system (JPK) and an inverted optical microscope (Olympus IX71) were used under liquid conditions in Standard solution with or without 2 mM cGMP. Rectangular silicon nitrite gold-coated cantilevers (HYDRA2R-50NGG from APPNANO) were functionalized and were used to localize plasma membrane patches and to perform SMFS experiments. For imaging of the membrane patch, the AFM system was operated in liquid using the tapping mode^44^ with ∼14 kHz as the operating frequency. The cantilever spring constant was ∼0.08 N m^−1^ and was calculated before the start of each experiment by using the equipartition theorem. A 0.4 numerical aperture/ × 10 objective was used to localize the area of oocyte incubation. The scan rate for AFM imaging was kept between 1 and 0.5 Hz, depending on the image size (from 20 to 0.5 μm) and on the condition of each sample. AFM images were acquired with a resolution of 512 pixels. Cantilever tip functionalization was based on the thiol and nitrilotriacetic acid-Ni^2+^ specificity for gold and the HisTag, respectively^68^,^69^. Tips were first cleaned in ethanol for 15 min, dried under an N_2_ flow and exposed to ultraviolet light for 15 min. The tips were further incubated for 5 min in chloroform and dried again under a N_2_ flow. These three steps were repeated one more time to obtain cleaner tips. Cantilevers were then incubated for 30 min in 10 μM dithiobis-C_2_-NTA (Dojingo Technologies, Japan), washed with ethanol and dried in a N_2_ flow. The tips were then incubated for 20 min in 100 μM NiSO_4_ and rinsed with MilliQ water before being dried in a N_2_ flow. Finally, the functionalized tips were incubated for 20 min in 10 mM 6-mercapto-1-hexanol to avoid nonspecific adsorption and were dried under a N_2_ flow. Salts and reagents were purchased from Sigma-Aldrich. To check the cantilever functionalization and activity of NTA, a control experiment was performed for each set of functionalized cantilevers. First, we performed an SMFS experiment on a polypeptide chain composed of eight modules of I27. The pulling efficiency was almost 10%, but in presence of 50, 100 and 200 mM imidazole the efficiency decreased to 1%, 0.5% and 0%, respectively (Supplementary Fig. 10).

### SMFS experiments and data processing

SMFS experiments were performed using membrane extracted from injected oocytes expressing constructs at a high level (about 1,000–5,000 channels per μm^2^) and in membrane extracted from uninjected oocytes as control. Oocytes were attached to a mica substrate or a gold surface (in case of cysteine mutant) and clean fragments of the membrane remained anchored to the substrate with the intracellular side exposed to the bathing medium and to the cantilever tip of the AFM. We used the AFM in liquid and in tapping mode^44^ to image the membrane patches. We observed membrane patches that had a high or a low protrusion density in their surface patterns (Supplementary Fig. 1h,i). In both cases, as would be expected from a membrane patch^44^, structures emerging from the mica had a height of 4–6 nm. Membrane patches with a high protrusion level had additional peaks that were 2–3 nm in height (Supplementary Fig. 1i) and were taken as suitable samples for SMFS experiments.

After the localization of the membrane patch, we moved the AFM tip over the imaged area (usually 1–2 μm^2^). Using the matrix scanning mode, the AFM tip was pushed into the surface with a contact force of 1 nN for 0.5 s, to give the protein a chance to adsorb on the stylus, and then retracted with a constant speed of 500 nm s^−1^, while the force exerted between the tip and surface was recorded. In ∼20% of the cases, the tip was able to adsorb a molecule, providing a sawtooth-like *F*–*D* curve, and if the magnitude of the force of these *F*–*D* curves was larger than 45 pN the curve was saved. In this manner, during an experimental session lasting up to 8–10 h, we collected ∼10,000 *F*–*D* curves for each experimental session, and a total of ∼200,000 and 300,000 *F*–*D* curves from membranes extracted from uninjected (control) oocytes in the presence and absence of cGMP, respectively; we also collected ∼300,000 and 450,000 *F*–*D* curves from membranes extracted from oocytes injected with mRNA for the CNGA1 channels in the absence and presence of cGMP, respectively. Approximately 30% of these *F*–*D* curves had only nonspecific adhesion events and the remaining 70% showed very diverse unfolding pathways, which could originate from the unfolding of endogenous proteins or the partial unfolding of CNGA1 channels. Therefore, we had to identify the *F*–*D* curves from the unfolding of the full CNGA1 channel and we had to distinguish these curves from all other unfolding events. We set up an initial filtering to remove the *F*–*D* curves showing only nonspecific adhesions and all other invalid *F*–*D* curves (see next section). Next, the remaining *F*–*D* curves were fitted to the worm-like chain (WLC) model^1^ with a persistence length (Lp) of 0.4 nm, and the corresponding contour length (Lc) was calculated using 0.4 nm as the length of a single a.a. All data points for Lc were summarized in histograms and fitted using Gaussian model. In text and in the figures, maxima of the Gaussian fittings are expressed as the mean±s.d.

### Initial filtering of *F*–*D* curves

The *F*–*D* curves that were collected by the AFM JPK software were filtered to remove unsuitable cases. Filtering was based on the analysis of the pushing (red curves) and pulling (black curves). First, retraction curves were treated by median and variance filtering (if the number of peaks in the filtered pulling curve was <2, the curve was discarded; Supplementary Fig. 2a). Force offset between the retraction and extension curves was compared and if the offset was greater than a given threshold related to the type of experiment (at least 30 pN, but usually is greater) in the initial part of the curve, the curve was discarded (Supplementary Fig. 2b). We then compared crossings between the extension and retraction curves and if the force offset between two crossings was >20 pN, the curve was discarded (Supplementary Fig. 2c). Variance *σ*^2^ of the extension curve was computed to estimate the motion of the baseline and if *σ*^2^ was >10 pN^2^, the curve was discarded (Supplementary Fig. 2d). Initial slope of the extension curve (Sp) and the retraction signal (Sr) was then compared and if (1−Sp/Sr) was >1, the curve was discarded (Supplementary Fig. 2d,e). The maximum pulling force (*F*) was calculated and if *F* was <30 pN, the curve was discarded (Supplementary Fig. 2f). The extension curve drift was calculated and if the maximum amplitude was >35 pN in the ‘flat’ region, the curve was discarded). Lengths of the TSS (distance or TSS) for the extension and retraction curves was compared and if the length difference was >10%, the curve was discarded. The (*F*,Lc) plot of the pulling curve was computed (the algorithm determines whether there is at least one peak, extracted as described in Methods, in the range between 80 and 300 nm and whether the last peak is in the range between 250 and 350 nm or a user-defined window; Supplementary Fig. 2a–f).

### Bioinformatics analysis

We performed bioinformatics to identify *F*–*D* curves from single subunit of CNGA1 channels. These *F*–*D* curves must be found only in SMFS experiments from injected oocytes and we have modified the existing algorithms^45^ for this purpose. The method had two steps. First, each *F*–*D* curve was mapped to a sequence of symbols that represented the location and amplitude of the force peaks (coding), and then these sequences were assembled in groups with similar properties (clustering). The coding step was based on the transformation of the *F*–*D* curves into a plot of force and contour lengths (*F*,Lc)^45^ (Fig. 1a–e). We used the WLC model where Lp is the persistence length (0.4 nm), a parameter that represents the stiffness of the molecule.

For each tip-sample separation (*D* or TSS) value, the WLC model is used to compute the corresponding value of Lc that is obtained by solving the third order polynomial: 4*λ*^3^+*ω λ*^2^−1=0, where *λ*=1−*D*/Lc and *ω*=4*F*(*D*,Lc)/*α*−3 and *α*=*k*_b_*T*/Lp. This equation has three roots and the root of interest is the real root λ* such that 0<λ*<1. In this manner, each point of the *F*–*D* curve (*F*,*D*) (for example, the curve in Fig. 1a) is transformed into a corresponding point (*F*,Lc), and each *F*–*D* curve is transformed into an (*F*,Lc) plot (see Fig. 1b). The three roots of the equation were obtained using a MATLAB routine. Owing to this transformation, each portion of the *F*–*D* curve that is fitted perfectly by the WLC model is mapped to a perfect vertical segment. The transformation of an *F*–*D* curve is therefore a relation (set of point) in the (*F*,Lc) plane rather than a function in the plane, and is also not a continuous curve.

Given a set of *F*–*D* curves, we computed the histogram of the normalized counts/bin of Lc values (normalized histogram of Lc values). The Lc axis of the (*F*, Lc) plot is first divided into bins (in the range from 1 to 10 nm). All points with a value of *F* larger than 30 pN are counted in the corresponding bin and summed over all sets of the *F*–*D* curves, and the final histogram of counts/bin is normalized so that its maximal value is equal to 1. This histogram is used to quantify the occurrence of points in the *F*–*D* curves that correspond to a given value of Lc.

Given an (*F*,Lc) plot, it is possible to extract an (*F*,Lc) profile and to compute the local maxima (Histogram of Lc values at force peaks). The Lc axis is divided into fixed intervals of 1–10 nm (typically of 5 nm). We extract the maximum value of *F* in each interval to obtain the corresponding (*F*,Lc) profile (if all values of *F* in an interval are below 30 pN, the value of the force in that interval is set equal to 0 pN) and the local maxima of the (*F*,Lc) profile are computed.

At this point, it is possible to compute the final histogram from the local maxima of the (*F*,Lc) profile that is normalized by the total number of *F*–*D* curves that were considered; in this way, the histogram of the local maxima shows the probability of obtaining *F*–*D* curves that have a force peak with a given value of Lc.

Three different increasingly complex coding schemes were considered. The simplest coding scheme (Coding I) only codes the location of force peaks and only considers the value of Lc neglecting the corresponding value of *F*. If the *F*–*D* curve has *n* force peaks with values of Lc and *F* equal to Lc_*i*_ and *F*_*i*_ for *i*=1,..*n* and having selected a bin width ΔLc (varying between 1 and 10 nm), each *F*–*D* curve is converted into a sequence of symbols of the type (0,0,1,0,0,0,1,...). All symbols are set to 0 with the exception of some values of 1 located at position *k* corresponding to the integer value of Lc_*i*_/ΔLc (see red circles in Fig. 1c). This coding scheme is a binary code and was considered for its simplicity. For the next scheme, given a bin width ΔLc, such as 5 or 10 nm, each *F*–*D* curve is converted into a sequence of symbols (0,0,F1,0,0,0,F2,...), where Fi is the value of the *k*_th_ force peak, and if Lck is the corresponding value of Lc, the symbol Fk is located at the position that corresponds to the integer value of Lck/ΔLc (Coding II and red circles in Fig. 1d). A more complex coding scheme (Coding III) considers all points in the (*F*,Lc) profiles with values of *F* larger than 30 pN; for each interval between *k*ΔLc and (*k*+1) ΔLc, this scheme selects the maximum value of the (*F*,Lc) plot in that interval (see red circles in Fig. 1e). In this case, the *F*–*D* curve is coded in the sequence of symbols *s*_1_,*s*_2_,*s*_3_,..*s*_*n*_, where *s*_*k*_ is the maximal value of *F* in the interval between *k*ΔLc and (*k*+1)ΔLc. Once the *F*–*D* curves are transformed into symbol sequences, a distance *D(x*_*i*_*,x*_*j*_) between the two sequences *x*_*i*_ and *x*_*j*_ must be defined.

We then applied different clustering procedures with the rationale that clusters containing *F*–*D* curves that only originate from membranes extracted from injected oocytes are ‘good’ candidates to represent the unfolding of CNGA1 channels. For the clustering step (Supplementary Note 2), we consider all *F*–*D* curves with the last peak with a value of Lc larger than 220 nm, which are obtained from membranes extracted from injected and uninjected oocytes, and we look for a cluster of similar *F*–*D* curves that are only obtained from injected oocytes. We used clustering methods developed in Computer Science, to identify objects or patterns with similar features. At the end of the informatics analysis, we identified three major clusters of similar *F*–*D* curves that were obtained only from membranes extracted from injected oocytes. Other clusters were nondiscriminatory and were composed of *F*–*D* curves obtained from membranes extracted from injected and uninjected oocytes.

We then developed an algorithm for obtaining the *F*–*D* curves. Most of the available clustering algorithms require the number of subsets/clusters *N* to be given and *N* is the critical parameter controlling the quality of the clusterization (Supplementary Fig. 4). We compared the performance of many clustering algorithms (Supplementary Fig. 3) for their ability to provide clusters with *F*–*D* curves only from injected oocytes and they performed similarly. The critical step of the proposed algorithm is the choice of the value of *N* providing homogeneous clusters of *F*–*D* curves only from injected oocytes. The algorithm for obtaining the *F*–*D* curves shown in Fig. 1f,g is composed of the following steps. In step 1 (initial filtering), all the obtained *F*–*D* curves were first filtered as described in the previous section, to remove bad *F*–*D* curves (Supplementary Fig. 2). In step 2 (second filtering according to their length), *F*–*D* curves that passed the initial filtering were further filtered according to the largest value of Lc. We have restricted our analysis to those *F*–*D* curves that had a maximum Lc value larger than 220 nm and only ∼1% of the *F*–*D* curves passed this filtering step. In step 3 (clustering), all *F*–*D* curves from injected (*S*_injected_) and uninjected oocytes (*S*_uninjected_) that passed step 1 and step 2 were merged in the same set *S*. *S* is composed by the union of *S*_injected_ and *S*_uninjected_. These *F*–*D* curves were coded as described before. The set *S* is broken in *N* subsets/clusters—with similar features—as described in details in the next section. In step 4 (choice of N), the value of *N* was progressively increased from 2 up to 300 and we searched for subsets/clusters containing only *F*–*D* curves from injected oocytes. The best value of *N* was chosen as the one generating the subset/cluster with the largest number of *F*–*D* curves containing only *F*–*D* curves from injected oocytes. The dependence of the clustering as a function of *N* is shown in Supplementary Fig. 4. Subsets/clusters of obtained *F*–*D* curves with similar features from injected oocytes are shown in Supplementary Fig. 5.

The same procedure was applied separately to *F*–*D* curves obtained in the absence of cGMP (Fig. 1) and in the presence of 2 mM cGMP (Fig. 2). In steps 1–4, *F*–*D* curves were not translated—or shifted—along the *x* axis—that is, the TSS.

In step 5, enrichment, *F*–*D* curves of the selected clusters *C*_*i*_ were taken as seeds of the ‘good’ *F*–*D* curves and enriched by comparison with all *F*–*D* curves belonging to *S*_injected_, which passed steps 1 and 2. *F*–*D* curves that were similar—allowing a horizontal shift of 5 nm—to those in *C*_*i*_ were added to *C*_*i*_.

### Clustering of *F*–*D* curves

Standard clustering procedures can be used after the *F*–*D* curves have been converted into symbol sequences *x*_1_,*x*_2_,.....*x*_n_ and a similarity matrix *Sim(x,y)* between the symbol sequences is available (see Supplementary Note 2). We have used and compared the average, centroid, complete, median, single, ward and weighted clustering algorithms (all these algorithms are agglomerative clustering algorithms in the MATLAB statistics toolbox http://www.mathworks.it/it/help/stats/clusterdata.html). For all these algorithms, the total number of clusters *N*_cluster_ must be assigned and the choice of *N*_cluster_ is crucial. We have circumvented this problem by mixing the *F*–*D* curves from membranes extracted from injected and uninjected oocytes, and we have varied the value of *N*_cluster_. For values of *N*_cluster_ that are <10, none of the tested clustering algorithms are able to distinguish between the *F*–*D* curves that were obtained from membranes extracted from injected and from uninjected oocytes. When *N*_cluster_ is between 50 and 100, clusters of *F*–*D* curves can be observed from injected oocytes with probabilities of 0.8, 0.9 and 1, that is, in which 80%, 90% and 100% of curves are from membranes extracted from injected oocytes (Supplementary Fig. 4). We found that when we used the clustering algorithm ‘complete’ and when *N*_cluster_ was larger than 100, several clusters from injected oocytes could be detected with a probability that was larger than 0.8. For the value of *N*_cluster_, we selected the value for which we had the largest clusters of *F*–*D* curves from membranes extracted from injected oocytes with a probability of 1. Examples of the obtained clusters are shown in the Supplementary Fig. 5. There are several clusters with *F*–*D* curves (Supplementary Fig. 5d–f) obtained from both injected (red curves) and uninjected oocytes (cyan curves). These *F*–*D* curves presumably represent the unfolding of proteins or molecules that form the plasma membrane of *X. laevis* oocytes and therefore can be obtained from either membranes extracted from injected or uninjected oocytes. As shown in Supplementary Fig. 5a, we found one cluster (Cluster 1-CS) with 22 *F*–*D* curves that were only obtained from injected oocytes. These *F*–*D* curves represent the seed of putative *F*–*D* curves obtained from the unfolding of CNGA1 channels from their C-terminal. Cluster 2-CS had *F*–*D* curves similar to those present in Cluster 1-CS and *F*–*D* curves of Cluster 3-CS had different force peaks.

The Cluster 1-CS of ‘good’ *F*–*D* curves obtained from the bioinformatics analysis were enriched in the following way: *F*–*D* traces of the selected clusters Cluster 1-CS are taken as seeds and are enriched by comparison with all *F*–*D* traces belonging to injected oocyte plasma membrane, which passed Steps 1 and 2 (see previous section). *F*–*D* traces, which were similar—allowing a horizontal shift of <5 nm—to those in Cluster 1-CS, were added to Cluster 1-CS. The used similarity measure consisted in the following criteria: the added *F*–*D* curve does not increase the intracluster distance described in the next section.

### Intracluster and intercluster similarity

Given a cluster C1 and the corresponding similarity matrix *Sim(x,y)* among the elements or sequences of C1, the intracluster difference IntraC_1_ is defined^70^ as: IntraC_1_=(1/(*n*^2^−1)) Σ_*i,j*_ *Sim(x*_*i*_*,x*_*j*_), where *n* is the total number of sequences in C1 and *x*_*i*_, *i*=1,...*n*, are the sequences in C1. Given two clusters C1 and C2, the intercluster difference InterC_1_C_2_ is defined^70^ as: InterC_1_C_2_=(1/*n m*) Σ_*j*_Σ_*i*_ *Sim(x*_*i*_*,y*_*j*_), where *n* and *m* are the total number of sequences in clusters *C*_1_ and *C*_2_, respectively, and *x*_*i*_ for *i*=1,...,*n* and *y*_*j*_ for *j*=1,…,*m* are the sequences present in *C*_1_ and *C*_2_, respectively. A cluster of fingerprints *f*_*n*_ FP identifies cluster *C*_*i*_ among a set of other clusters *C*_*k*_, *k*=,…,*N*, if InterFPC_*i*_<InterFPC_*k*_ for all *k* different from *i*. A cluster of finger prints *f*_*n*_ FP identifies cluster *C*_*i*_ very well among a set of clusters *C*_*k*_, *k*=,…,*N*, if InterFPC_*i*_<InterFPC_*k*_ for all *k* different from I and if InterFPC_*i*_ is very similar (within 10%) to IntraC_*i*_ and IntraFP. In Fig. 1f of the main text, the set of fingerprints with the N2B construct identifies cluster 1 among all clusters that were obtained as described below according to all considered codings and similarities, and identifies cluster 1 very well according to coding I. The *F*–*D* curves from the CNGA1-N2B-HisTag construct have slightly higher forces; thus, according to codings II and III, the value of InterFP clusters using the MAE method is higher for IntraFP clusters and approximately twice the value of Intra-clusters, whereas using the Hamming-force method, InterFP clusters is higher for IntraFP clusters and for Intra clusters (see Supplementary Table 2).

In Fig. 1f, clusters were obtained using Ncluster=200 and by selecting those clusters in which at least 80% of the curves were obtained from membranes extracted from injected oocytes. Using this procedure, we identified 15 clusters and 3 were obtained only from membranes extracted from injected oocytes. *F*–*D* curves with the N2B fingerprint identified cluster I as obtained from the unfolding of CNGA1 channels. Supplementary Table 6 reports the Intracluster similarity for the three clusters and for the set of *F*–*D* curves (FP) with the N2B fingerprint, and also shows the Intercluster similarity between each cluster and FP.

## Additional information

**How to cite this article:** Maity, S. *et al.* Conformational rearrangements in the transmembrane domain of CNGA1 channels revealed by single-molecule force spectroscopy. *Nat. Commun.* 6:7093 doi: 10.1038/ncomms8093 (2015).

## Supplementary Material

Supplementary Information Supplementary Figures 1-10, Supplementary Tables 1-2, Supplementary Notes 1-4 and Supplementary References
