# Corrigendum: Conformational rearrangements in the transmembrane domain of CNGA1 channels revealed by single-molecule force spectroscopy

**DOI:** 10.1038/ncomms16162

**Published:** 2017-09-29

**Authors:** Sourav Maity, Monica Mazzolini, Manuel Arcangeletti, Alejandro Valbuena, Paolo Fabris, Marco Lazzarino, Vincent Torre

Nature Communications 6: Article number: 7093 ; DOI: 10.1038/ncomms8093 (2015); Published: 05 12 2015; Updated: 09 29 2017

An error was inadvertently introduced into the fifth sentence of the Abstract in the original version of this Article. The sentence should have stated ‘Force spectra determined that the S4 transmembrane domain is mechanically coupled to S5 in the open state, but S3 in the closed state’ and not ‘Force spectra determined that the S4 transmembrane domain is mechanically coupled to S5 in the closed state, but S3 in the open state’. This has now been corrected in both the PDF and HTML versions of the Article.

