# Supplementary material for: Conformational rearrangements in the transmembrane domain of CNGA1 channels revealed by single-molecule force spectroscopy
Source: Nat Commun. 2015 May 12;6:7093. doi: 10.1038/ncomms8093 (PMC4432583; doi:10.1038/ncomms8093)
Supplement: Supplementary Information — Supplementary Figures 1-10, Supplementary Tables 1-2, Supplementary Notes 1-4 and Supplementary References [file ncomms8093-s1.pdf]

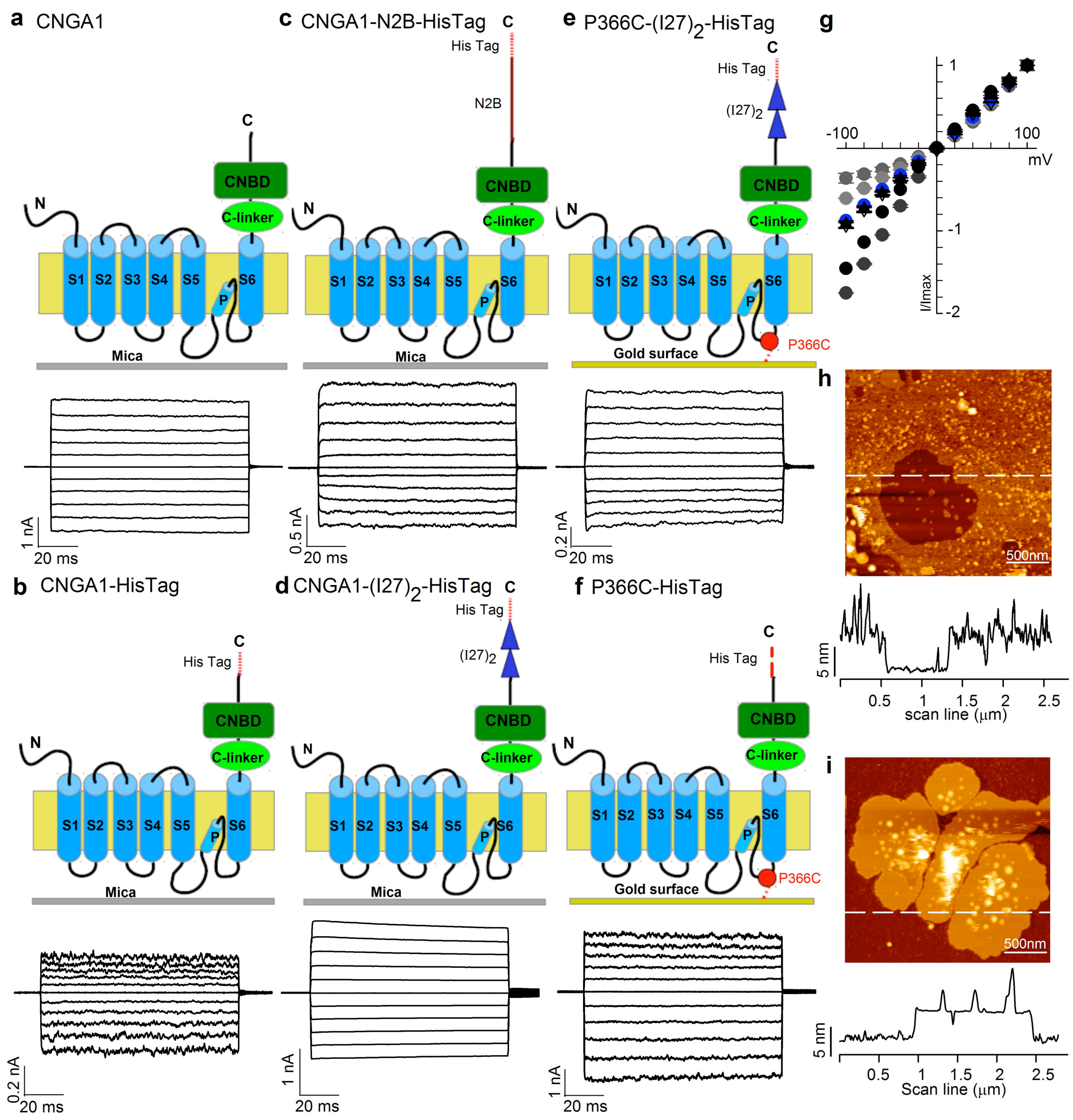

Supplementary Figure 1: The constructs and fingerprints that were utilized. a-f: schematic representation (upper panel) and the corresponding current recordings (lower panel) of the CNGA1 channel (**a**), CNGA1-HisTag (**b**), CNGA1-N2B-HisTag (**c**), CNGA1-$(I27)_2$-HisTag (**d**), P366C-$(I27)_2$-HisTag (**e**) and P366C-HisTag (**f**). For current recordings of the CNGA1-CNGA1 tandem, see Ref. 1. The current recordings are measured in an inside-out excised patch under voltage-clamp conditions; the voltage steps are from -100 to 100 mV. The curves are obtained from the average of at least 5 recordings in the presence of 2 mM cGMP minus the average of at least 2 recordings in the closed state. **g:** I/V relationships of the cGMP-activated current from the CNGA1 channel (blue circles), the CNGA1-HisTag construct (dark gray circles), the CNGA1-N2B-HisTag construct (gray circles), the CNGA1-$(I27)_2$-HisTag construct (white triangles), the P366C-$(I27)_2$-HisTag construct (black triangles) and the mutant channel P366C-HisTag (middle gray circles). **h-i:** Representative AFM images of membrane patches with a high or a low protrusion density with the corresponding height profile taken along the dashed lines.

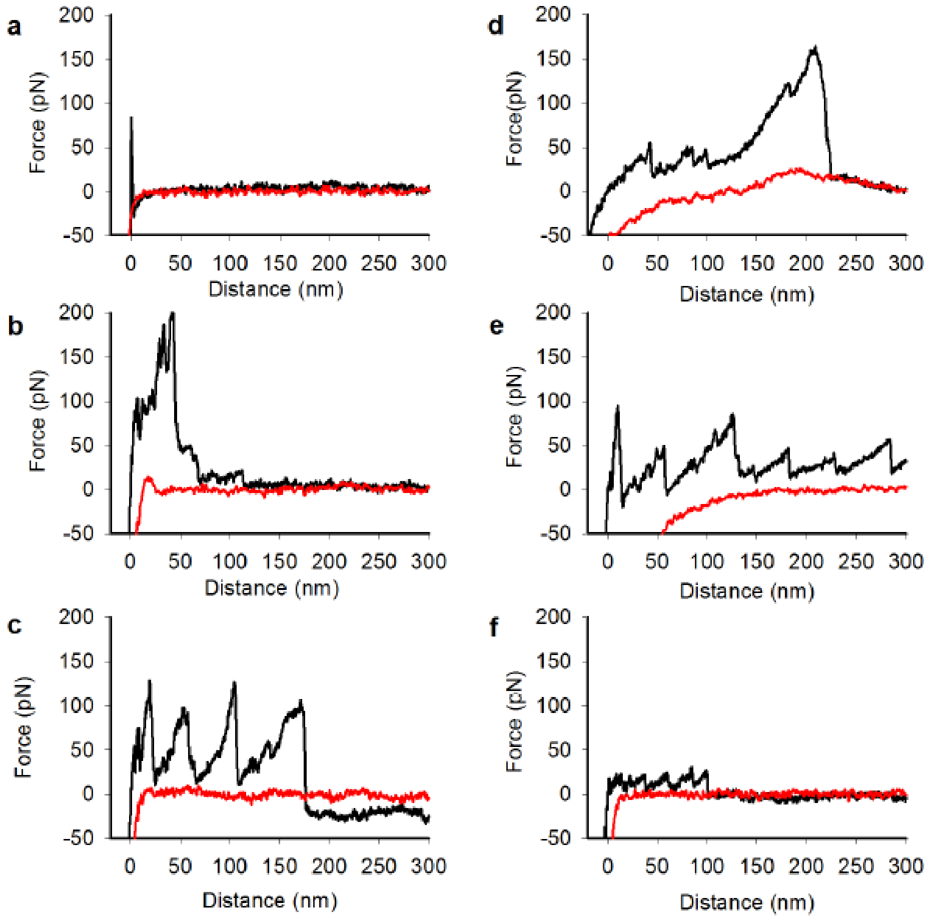

Supplementary Figure 2: Examples of F-D curves that were discarded by our filtering steps. **a:** absence of a force and a tip-sample separation (distance or TSS) that demonstrate the absence of an interaction between the cantilever and the protein. **b:** high initial nonspecific force that is most likely due to interactions with the oocyte membrane surface; **c:** short tip-sample separation showing an Lc value that does not correspond to our constructs; **d:** curve representing the attachment of aggregate to the tip showing artifact; **e:** curve showing not complete unfolding at the cantilever full extension; **f:** low force and short tip-sample separation that are not characteristic of our constructs. The tip-sample separation for the approach of the cantilever to the surface are shown in red, and the data for the retraction are shown in black.

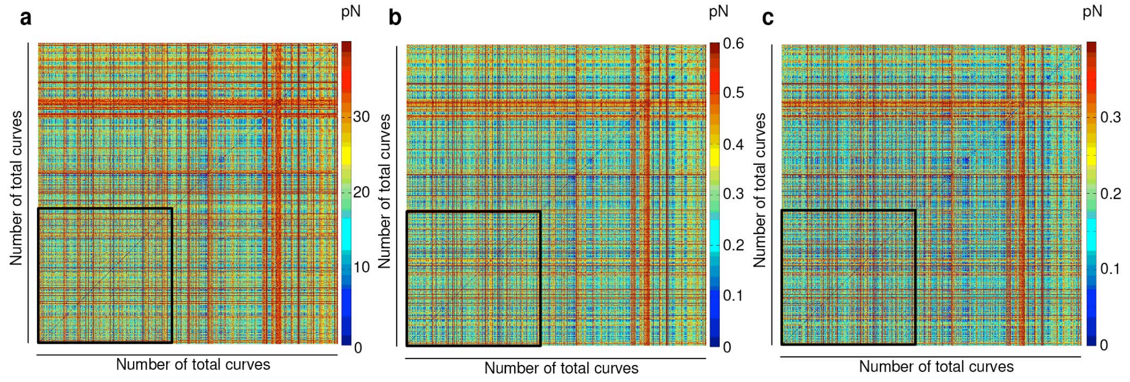

Supplementary Figure 3: The similarity matrix. Examples of the similarity matrix between 1585 traces which passed our filtering step. The matrix is symmetric and has 1585x1585 entries. Each entry of the matrix represent: **a:** MAE method, **b:** Hamming method, **c:** Binary method. Blue represents the minimum value indicating a high similarity among F-D curves and red indicates that the corresponding F-D curves are very different. The black box represents the F-D curves obtained from membranes extracted only from injected oocytes.

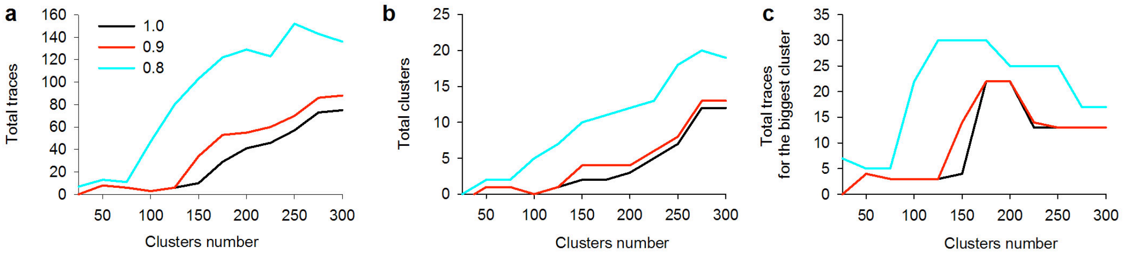

Supplementary Figure 4: Clustering of F-D curves as a function of $N_{cluster}$ a: relation between the total number of F-D curves from membranes extracted from injected oocytes with probabilities of 0.8, 0.9 and 1 as a function of $N_{cluster}$. **b:** relationship between the total number of clusters from membranes extracted from injected oocytes with probabilities of 0.8, 0.9 and 1 as a function of $N_{cluster}$. **c:** relationship between the total number of F-D curves of the largest cluster from membranes extracted from injected oocytes with probabilities of 0.8, 0.9 and 1 as a function of $N_{cluster}$. Clustering was obtained using the "complete" algorithm and the MAE distance. As shown in (**c**), when $N_{cluster}$ is approximately 200 and clusters with more than 20 F-D curves obtained from injected oocytes aree observed. Clustering was therefore performed with a value of $N_{cluster}$ equal to 200.

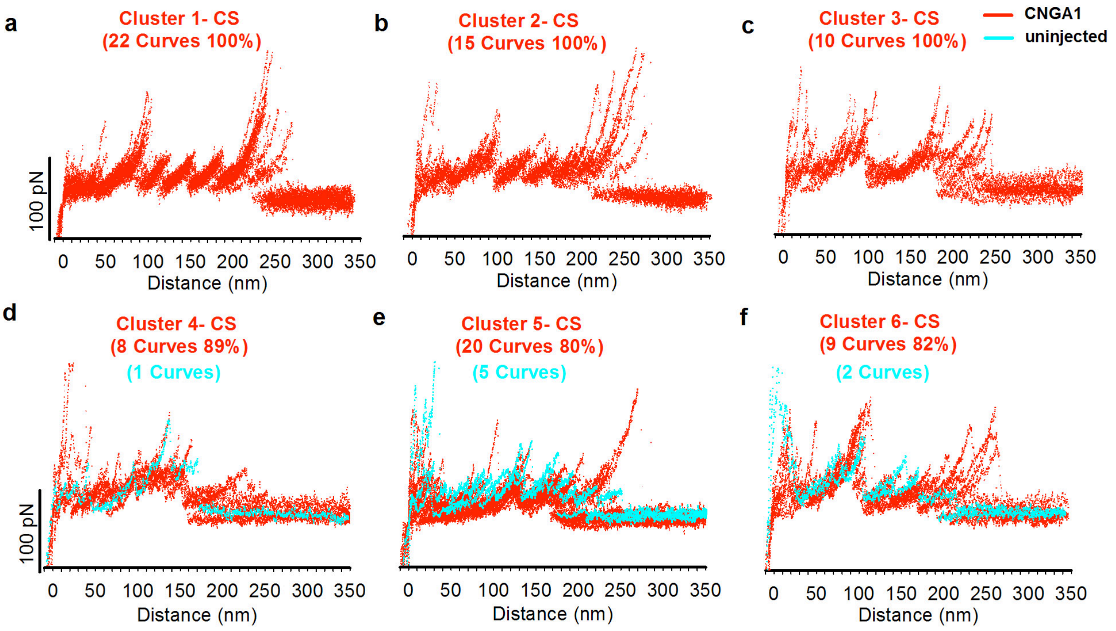

Supplementary Figure 5: Clustering of F-D curves: a-c: three different clusters containing curves from membranes extracted from injected oocytes for CNGA1 channels in the closed state. **d-f:** example of clusters containing curves from membranes extracted from injected (red) and uninjected (cyan) oocytes.

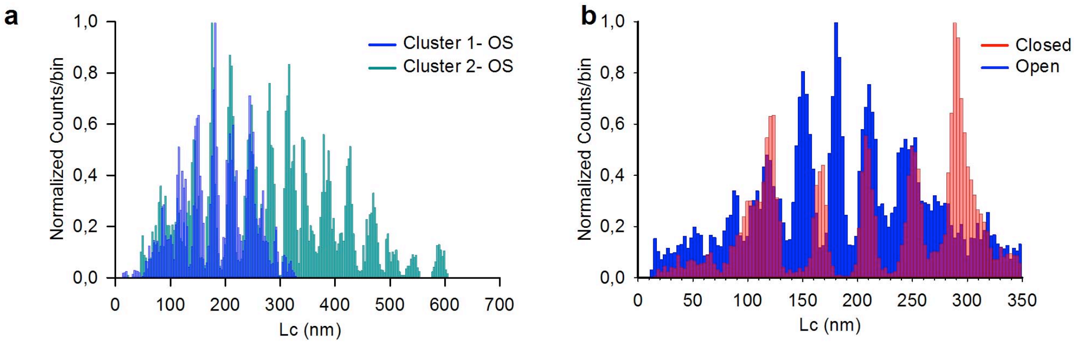

Supplementary Figure 6: Two clusters of CNGA1 channels in the open state and comparison between open and closed states. a: superimposition of the histograms of the normalized counts/bin against Lc (see Methods) from the F-D curves from the unfolding of a single (blue) and two concatenated (cyan) CNGA1 channels in the open state. **b:** superposition of the histograms of the normalized counts/bin compared with Lc for CNGA1 in the open state (blue) and the closed state (red).

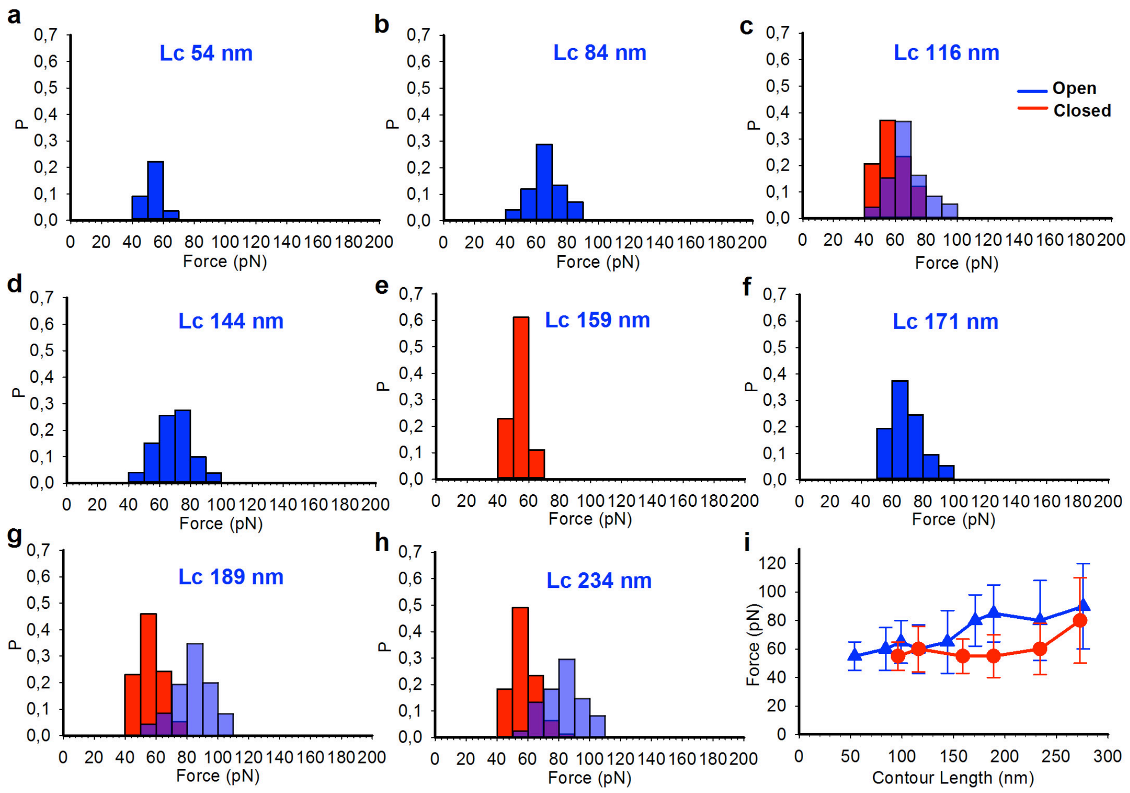

Supplementary Figure 7: Force distribution histograms. a-h: histograms corresponding to the different Lc values for 157 F-D curves from the CNGA1 construct in the closed state (red) and 132 F-D curves in the open (blue) state. **i:** plot comparison between the force in the closed (n=157; red) and open (n=132; blue) states; mean±S.D.

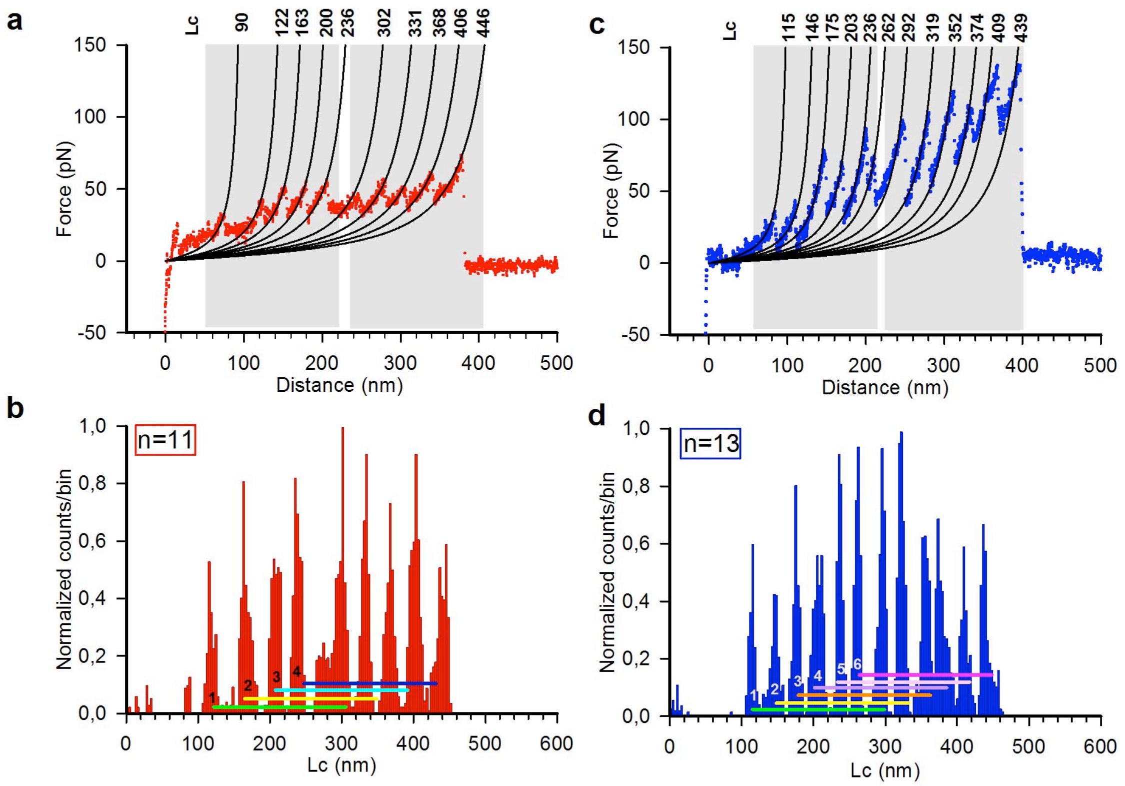

Supplementary Figure 8: F-D curves from the CNGA1-CNGA1 tandem construct in the closed and open states. a,c: F-D curves recorded during the unfolding of a single construct in the closed state (red) and in the open state (blue). **b,d:** histograms of the normalized counts/bin against Lc (see Methods) from the F-D curves of panel (**a**) (red) and the F-D curves of panel (**c**) (blue) in the closed and in the open states, respectively. The colored lines (180 nm) indicate that these repeats are separated by approximately 180±6 nm.

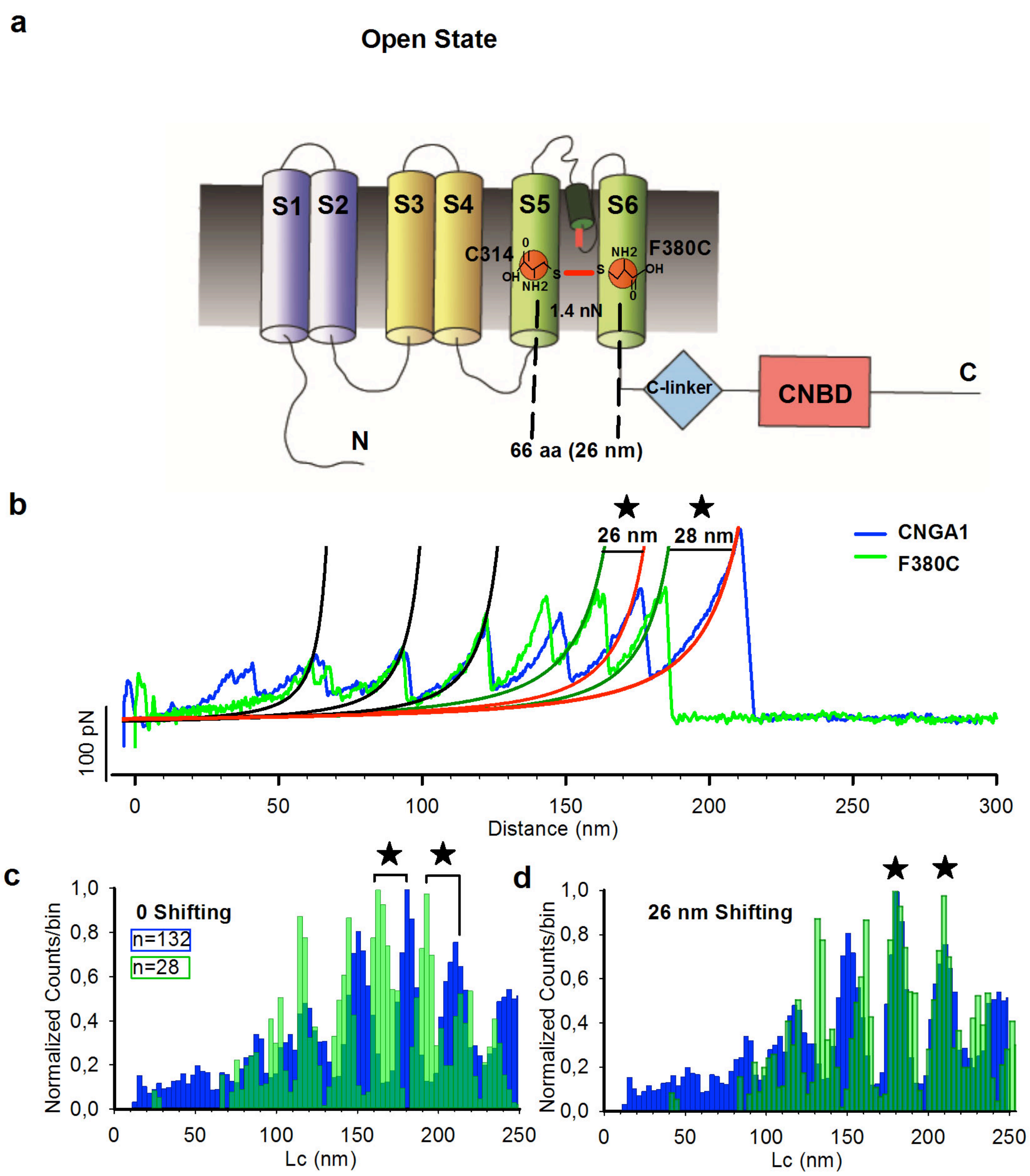

Supplementary Figure 9: Unfolding of the mutant channel F380C in the open state. a: schematic representation of the interaction between the endogenous C314 and the exogenous cysteine at position 380 in the mutant channel F380C. **b:** superposition of a representative F-D curve from CNGA1 (blue) and a curve from the mutant channel F380C (green) in the presence of 2 mM cGMP; the F-D curve from the mutant channel F380C was obtained in the presence of 10 µm CuP to promote the formation of S-S bonds. The F-D curves were fitted using the WLC model. The first three force peaks have very similar values of Lc, whereas the last two force peaks (indicated by the black star) of mutant channel F380C are shifted to the left by 26 nm, corresponding to the full extension for stretching the a.a. from C314 to C380. **c:** superimposition histogram representing the normalized frequency of events versus the Lc for the CNGA1 channel (blue) and mutant channel F380C (green). **d:** the same as in d but with a shift of 26 nm.

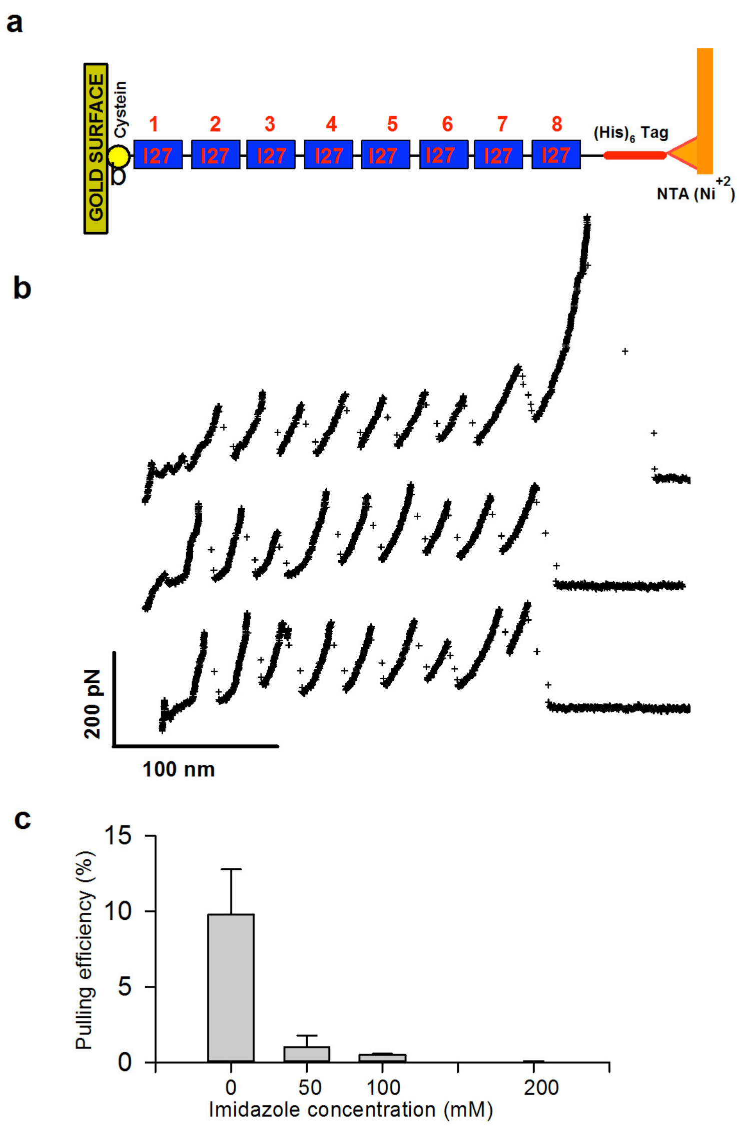

Supplementary Figure 10: Blocking experiments for NTA-functionalized cantilever. a: schematic representation of the I27 constructs composed by 8 modules; the N-termini of the first module contain a cysteine while the C-termini of the last module contain a HisTag composed by 6 residues. In the scheme are also represented the gold surface that bind the cysteine and the functionalized NTA-cantilever binds to the HisTag. **b:** examples F-D curves obtained at 0 mM imidazole in PBS. **c:** Comparison of pulling efficiency at different concentration of imidazole in PBS.

**Oligonucleotide name**	**Sequence (in base pair)**
HisTag Forward	TGGGCCCACACATCATCACCATCATCACGACTCTACACA<br>GG
HisTag Reverse	GTGAGCTGATACCGCTCGCCG
ApaI Forward	GGAACTGAAAGTGGTCCCACAGACTCTAC
ApaI Reverse	GTAGAGTCTGTGGGACCACTTTCAGTTCC
Linker Forward	GTGGGCCCACAGACTCTACACAGGACGGAAGCGGAGGAA<br>CCGAGTTGGGAAGTACCATGAAGAAAGTGATTATCAATA<br>CATGG
Linker Reverse	CCATTCGGGTGTTCTTGAGGCTG
P366C Forward	GGCGAAACACCATGTCCTGTGAGGGATTCT
P366C Reverse	AGAATCCCTCACAGGACATGGTGTTTCGCC
F380C Forward	TTTGTGGTGGCTGATTGCCTCATTGGAGTGTTAATT
F380C Reverse	AATTAACACTCCAATGAGGCAATCAGCCACCACAAA
P293A Forward	GAAACACGGACAAACTACGCAAACATCTTCAGGATCTC
P293A Reverse	GAGATCCTGAAGATGTTTGCGTAGTTTGTCCGTGTTTC
**Construct sequence**	**Sequence (in a.a.)**
CNGA1	MKKVIINTWHSFVNIPNVVGPDVEKEITRMENGACSSFS<br>GDDDDSASMFEESETENPHARDSFRSNTHGSGQPSQREQ<br>YLPGAIALFNVNNSSNKEQEPKEKKKKKKEKKSKPDDKN<br>ENKKDPEKKKKKEKDKDKKKKEEKGKDKKEEEKKEVVVI<br>DPSGNTYYNWLFCITLPVMYNWTMIIARACFDELQSDYL<br>EYWLAFDYLSDVVYLLDMFVRTRTGYLEQGLLVKEERKL<br>IDKYKSTFQFKLDVLSVIPTDLLYIKFGWNYPEIRLNRL<br>LRISRMFEFFQRTETRTNYPNIFRISNLVMYIIIIIHWN<br>ACVYFSISKAIGFGNDTWVYPDVNDPDFGRLARKYVYSL<br>YWSTLTLTTIGETPPPVRDSEYFFVVADFLIGVLIFATI<br>VGNIGSMISNMNAARAEFQARIDAIKQYMHFRNVSKDME<br>KRVIKWFDYLWTNKKTVDEREVLKYLPDKLRAEIAINVH<br>LDTLKKVRIFADCEAGLLVELVLKLQPQVYSPGDYICKK<br>GDIGREMYIIKEGKLAVVADDGITQFVVLSDGSYFGEIS<br>ILNIKGSKAGNRRTANIKSIGYSDLFCLSKDDLMEALTE<br>YPDAKGMLEEKGKQILMKDGLLDINIANAGSDPKDLEEK<br>VTRMESSVDLLQTRFARILAEYESMQQKLKQRLTKVEKF<br>LKPLIDTEFSAIEGSGTESGPTDSTQDGGRSSRTRGG
CNGA1-N2B-HisTag	MKKVIINTWHSFVNIPNVVGPDVEKEITRMENGACSSFS<br>GDDDDSASMFEESETENPHARDSFRSNTHGSGQPSQREQ<br>YLPGAIALFNVNNSSNKEQEPKEKKKKKKEKKSKPDDKN<br>ENKKDPEKKKKKEKDKDKKKKEEKGKDKKEEEKKEVVVI<br>DPSGNTYYNWLFCITLPVMYNWTMIIARACFDELQSDYL<br>EYWLAFDYLSDVVYLLDMFVRTRTGYLEQGLLVKEERKL<br>IDKYKSTFQFKLDVLSVIPTDLLYIKFGWNYPEIRLNRL<br>LRISRMFEFFQRTETRTNYPNIFRISNLVMYIIIIIHWN<br>ACVYFSISKAIGFGNDTWVYPDVNDPDFGRLARKYVYSL<br>YWSTLTLTTIGETPPPVRDSEYFFVVADFLIGVLIFATI<br>VGNIGSMISNMNAARAEFQARIDAIKQYMHFRNVSKDME<br>KRVIKWFDYLWTNKKTVDEREVLKYLPDKLRAEIAINVH<br>LDTLKKVRIFADCEAGLLVELVLKLQPQVYSPGDYICKK<br>GDIGREMYIIKEGKLAVVADDGITQFVVLSDGSYFGEIS<br>ILNIKGSKAGNRRTANIKSIGYSDLFCLSKDDLMEALTE

	YPDAKGMLEEKGKQILMKDGLLDINIANAGSDPKDLEEK<br>VTRMESSVDLLQTRFARILAEYESMQQKLKQRLTKVEKF<br>LKPLIDTEFSAIEGSGTESGPTDSTQDGGRSSRDTEKIF<br>PSAMSIEQINSLTVEPLKTLLAEPEGNYPQSSIEPPMHS<br>YLTSVAEEVLSPKEKTVSDTNREQRVTLQKQEAQSALIL<br>SQSLAEGHVESLQSPDVMISQVNYEPLVPSEHSCTEGGK<br>ILIESANPLENAGQDSAVRIEEGKSLRFPLALEEKQVLL<br>KEEHSDNVVMPPDQIIESKREPVAIKKVQEVQGRDLLSK<br>ESLTRGGHHHHHH
CNGA1-(I27)$_2$-HisTag	MKKVIINTWHSFVNIPNVVGPDVEKEITRMENGACSSFS<br>GDDDDSASMFEESETENPHARDSFRSNTHGSGQPSQREQ<br>YLPGAIALFNVNNSSNKEQEPKEKKKKKKEKKSKPDDKN<br>ENKKDPEKKKKKEKDKDKKKKEEKGKDKKEEEKKEVVVI<br>DPSGNTYYNWLFCITLPVMYNWTMIIARACFDELQSDYL<br>EYWLAFDYLSDVVYLLDMFVRTRTGYLEQGLLVKEERKL<br>IDKYKSTFQFKLDVLSVIPTDLLYIKFGWNYPEIRLNRL<br>LRISRMFEFFQRTETRTNYPNIFRISNLVMYIIIIIHWN<br>ACVYFSISKAIGFGNDTWVYPDVNDPDFGRLARKYVYSL<br>YWSTLTLTTIGETPPPVRDSEYFFVVADFLIGVLIFATI<br>VGNIGSMISNMNAARAEFQARIDAIKQYMHFRNVSKDME<br>KRVIKWFDYLWTNKKTVDEREVLKYLPDKLRAEIAINVH<br>LDTLKKVRIFADCEAGLLVELVLKLQPQVYSPGDYICKK<br>GDIGREMYIIKEGKLAVVADDGITQFVVLSDGSYFGEIS<br>ILNIKGSKAGNRRTANIKSIGYSDLFCLSKDDLMEALTE<br>YPDAKGMLEEKGKQILMKDGLLDINIANAGSDPKDLEEK<br>VTRMESSVDLLQTRFARILAEYESMQQKLKQRLTKVEKF<br>LKPLIDTEFSAIEGSGTESGPTDSTQDTRSGNTSGPGSR<br>TGLIEVEKPLYGVEVFVGETAHFEIELSEPDVHGQWKLK<br>GQPLAASPDCEIIEDGKKHILILHNCQLGMTGEVSFQAA<br>NTKSAANLKVKELRSVDLIEVEKPLYGVEVFVGETAHFE<br>IELSEPDVHGQWKLKGQPLAASPDCEIIEDGKKHILILH<br>NCQLGMTGEVSFQAANTKSAANLKVKELRSTGHHHHHH

Supplementary Table 1: Oligonucleotide primer sequences and a.a. sequence of the principal constructs that were utilized for the experiments. In the table are presented the oligonucleotides used for the preparation of mutations (P366C, F380C, P293A) in the CNGA1 sequence and the cloning of CNGA1 tandem (linker and relative mutations). CNGA1 is composed by with 690 a.a. residues (in black). The constructs CNGA1-N2B-HisTag and CNGA1-(I27)$_2$-HisTag contain a tag composed by 6 histidines (named HisTag in cyan) and a sequence of N2B (in green) or two modules of I27 (in red) at the C-terminus.

Coding	Method	Cluster	Intracluster	Intra FP	Intercluster (with FP)
I	Hamming	1	0.34	0.32	0.33
I	Hamming	2	0.48	0.32	0.57
I	Hamming	3	0.42	0.32	0.95
II	Hamming-force	1	2.35e-10	3.16e-10	3.34e-10
II	Hamming-force	2	2.22e-10	3.16e-10	3.53e-10
II	Hamming-force	3	2.92e-10	3.16e-10	3.89e-10
III	MAE	1	1.24e-11	2.12e-11	2.27e-11
III	MAE	2	9.67e-12	2.12e-11	2.35e-11
III	MAE	3	1.38e-11	2.12e-11	2.68e-11

Supplementary Table 2: Intercluster and intracluster similarity. The unit for Hamming-force and MAE is in Newton (N) while for the Hamming is a percentage (%).

Supplementary Note 1: CNGA1 constructs expression

Oocytes that had been injected with the construct CNGA1-HisTag (Supplementary Fig.1b) had a lower current respect to the current obtained with the construct CNGA1 (Supplementary Fig.1a) probably because a lower expression of the protein on the plasma membrane, or because the HisTag, directly attached to the C-terminus, can easily interact with some domain of the channel. The cGMP-activated currents were slightly inward-rectifying, and the mutant F380C channels showed significant rectification, as previously described[2]. Pulling experiments with the construct CNGA1-$(I27)_2$-HisTag did not produce F-D curves that contained the unfolding of the CNGA1 channels or the sawtooth pattern that is attributable to the unfolding of the I27 modules. The reason for this failure is that the force necessary to unfold the I27 modules is approximately 200 pN, which is significantly larger than the force required to unfold CNGA1 channels (see main text Figure 4). The construct CNGA1-$(I27)_2$-HisTag could therefore be pulled away from the membrane without unfolding the I27 modules. Only the experiments obtained with the construct P366C-$(I27)_2$-HisTag are reported in the main text and are illustrated in Fig.3.

Supplementary Note 2: Coding of F-D curves and similarity between them.

Computer Science and pattern recognition methods for clustering objects or instances with similar features require a measure of their similarity. To determine the similarity between different F-D curves, we coded F-D curves as an appropriate sequence of symbols, and we constructed similarity measures between the coded F-D curves. The coding was based on the observation that the F-D curves obtained in SMFS experiments are well fitted by the superposition of curves obtained from the WLC model (see Methods). We used three different methods to compute the similarity: the classic MAE (mean absolute error) method, the Hamming method and the binary-force method. These methods are used with the different coding schemes. MAE is used with Coding III (F,Lc), and Hamming-binary is used with Coding I and II.
The similarity based on the MAE is expressed in force (pN) and is computed as:

$$MAE = 1/n \sum_{k=1}^{n} \left| x_i(k) - x_j(k) \right|$$

Where n is the total number of points, and x_i and x_j are the coded sequence of symbols.
The Hamming-force similarity is computed as:

$$Hamming - force = \sum_{k=1}^{n} \left| x_i(k) - x_j(k) \right|$$

where n is the total number of points, and x_i and x_j are the coded sequence of symbols.
For the binary force, each peak of the F-D curves is individually compared with every other peak in a selected curve. If the shift of the peak (in bin size), along the Lc, is in the range [-1, 0, 1], the error is computed as for the Hamming-force. Otherwise, the error is set to zero. The similarity matrices *Sim(x,y)* that were used are shown in Supplementary Fig.3a-c. A low value of the entry of these matrices indicates a high similarity among the corresponding F-D curves.

Supplementary Note 3: SMFS of the CNGA1-CNGA1 tandem.

To further validate that the F-D curves of Fig.1f and Fig.2b are obtained from the unfolding of CNGA1 channels in the closed and open states, respectively, we performed SMFS on the CNGA1-CNGA1 tandem construct obtained from the concatenation of two identical CNGA1 subunits linked

by a short linker (10 a.a. between the C-terminal end of the first subunit and the N-terminal end of the second subunit). When the mRNA of the CNGA1-CNGA1 tandem construct is injected into oocytes, there is good expression of functional channels with properties very similar to those of CNGA1 channels[3]. The F-D curves obtained from the unfolding of the CNGA1-CNGA1 tandem are expected to have the largest value of Lc (above 400 nm) and to have well-defined repeats of force peaks separated by an appropriate value of ΔLc.

Each CNGA1 subunit contains seven endogenous cysteines: C35, C169, C186, C314, C481, C505 and C573. C35 near the N-terminus is thought to interact with C481 in the open state but not in the closed state[4,5]. C169 and C186 are in S1, and C314 is located in S5 and is known to interact with residues at position 380 in the S6 domain[6]. C481 and C505 are located in the C-linker and in the CNB domain, respectively[7]. C573 is located near the C-terminus of the channel[3] and does not seem to have any structural or functional role. In the absence of the formation of C-C bonds, unfolding of the second subunit will be repeated at a distance corresponding to 690+10 a.a., i.e., approximately 280 nm. However, in the presence of a C-C bond between C35 and C481, which is promoted by the interaction of the two subunits, the distance between the two repeats will be shorter by approximately 102 nm, i.e., 178 nm.

We performed SMFS experiments with the CNGA1-CNGA1 tandem construct, and in the closed state, we obtained 11 F-D curves with clear force repeats (Supplementary Fig.8a). The analysis of Lc histogram indicated that these repeats were separated by approximately 180±6 nm (Supplementary Fig.8b). In the open state, we obtained 13 F-D curves with a repeated pattern (Supplementary Fig.8c,d) very similar to that observed for the unfolding of CNGA1 channels in the open state (Fig.2). The force peaks from the tandem constructs in the open state were larger, varying from 50 to 140 pN, than those obtained in its absence, in agreement with the results for CNGA1 channels (compare Figs.1-2).

Supplementary Note 4: SMFS of the mutant channel F380C.

An additional test for the validation of the F-D curves attributed to the unfolding of CNGA1 channels is the measurement of the unfolding of the mutant channel F380C (Supplementary Fig.9a). In the presence of cGMP, this channel is locked in the open configuration by the formation of an S-S bond between the native C314 and the exogenous cysteine at position 380 (Ref. 6). Because an S-S bond has a breaking force of approximately 1.4 nN (Ref. 8) and CNGA1 channels have a mechanical stability below 150 pN, it is obvious that we unfold the CNGA1 channel without breaking the S-S bond. In the presence of an S-S bond, the total stretched length decreases by $N_{a.a.} \cdot l_{a.a.}$, where $N_{a.a.}$ is the total number of a.a. in the S-S loop, and $l_{a.a.}$ is the mean length of a single amino acid, which is assumed to be 0.4 nm (Ref. 9). Consequently, the F-D curves from the mutant channel F380C are expected to be shorter by 66 a.a. (F380-C314), which corresponds to an Lc of ~26 nm (Supplementary Fig.9a). We performed SMFS on the mutant channel F380C in the presence of 2 mM cGMP with 10 μm CuP, an agent that promotes the formation of S-S bonds[6]. We used the F-D curves obtained from the CNGA1 channels in the open state (Fig.2) as a template to identify the F-D curves obtained from the unfolding of the mutant channel F380C.

We identified 28 curves that when superimposed and aligned had initial force peaks corresponding to those of the CNGA1 channels (Supplementary Fig.9b): the F-D curves from the mutant channel F380C (green curves) superimposed on those from the CNGA1 channels (blue curves) and had common force peaks with Lc values of approximately 54, 86 and 116 nm (black WLC curves in Fig.SI9b). The force peaks observed in CNGA1 channels with values of 189±5 (mean±S.D., n=28) and 234±6 (mean±S.D., n=28) nm (red WLC curves in Supplementary Fig.9b) in the mutant channel F380C appear to be shifted by approximately 26-28 nm to the left (green WLC curves in Supplementary Fig.9b). These results indicate that the F-D curves from the mutant channel F380C have a gap of 26±2 (mean±S.D., n=28) nm compared with those from the CNGA1 channels. Inspection of the Lc histograms shows a similar result: the initial and final portions of the F-D

curves from the mutant channel F380C and the CNGA1 channel have the force peaks with the same value of Lc, provided that approximately 66 amino acids are deleted (Supplementary Fig.9c,d). If the mutant channel F380C in the open state has a disulfide bond between the endogenous C314 and the exogenous cysteine at position 380, the F-D curves obtained from its unfolding are expected to be 26.4 nm shorter than those obtained from the CNGA1 channels, in agreement with the experimentally observed gap of 26±2 nm (Supplementary Fig.9c,d). In fact, our results provide an experimental validation of the identification of F-D curves of Figs.1-2. Because the same conformational changes are detected both in electrophysiological[6] and SMFS experiments, we are assured that we are detecting functional conformational changes of CNGA1 channels also when the plasma membrane was attached to the surface.

Supplementary References

1-Mazzolini, M., Nair, a. & Torre, v. A comparison of electrophysiological properties of the CNGA1, CNGA1tandem and CNGA1cys-free channels. *European biophysics journal* 947-59 (2008).

2-Nair, A.V., Mazzolini, M., Codega, P., Giorgetti, A. & Torre, V. Locking CNGA1 channels in the open and closed state. *Biophys J.* **90,** 3599-607 (2006).

3-Mazzolini, M., Nair, A, & Torre, V. A comparison of electrophysiological properties of the CNGA1, CNGA1tandem and CNGA1cys-free Channels. *Eur. Biophys. J.* **37,** 947-959 (2008).

4- Rosenbaum, T. & Gordon, S.E. Dissecting intersubunit contacts in cyclic nucleotide-gated ion channels. *Neuron* **33,** 703-713 (2002).

5- Gordon, S.E., Varnum, M.D. & Zagotta, W.N. Direct interaction between amino- and carboxyl-terminal domains of cyclic nucleotide-gated channels. *Neuron* **19,** 431-441 (1997).

6- Nair, A.V., Mazzolini, M., Codega, P., Giorgetti, A. & Torre, V. Locking CNGA1 channels in the open and closed state. *Biophys. J.* **90,** 3599-3607 (2006).

7- Nair, A.V., Anselmi, C. & Mazzolini, M. Movements of native C505 during channel gating in CNGA1 channels. *Eur. Biophys. J.* **38,** 465-478 (2009).

8- Grandbois, M., Beyer, M., Rief, M., Clausen-Schaumann, H. & Gaub, H.E. How strong is a covalent bond? *Science* **283,** 1727-1730 (1999).

9- Rief, M., Gautel, M., Oesterhelt, F., Fernandez, J.M. & Gaub, H.E. Reversible unfolding of individual titin immunoglobulin domains by AFM. *Science* **276**, 1109-1112 (1997).
